# A sstR2-targeted radiohybrid theranostic agent for PET imaging and β^-^ therapy with excellent preclinical performance

**DOI:** 10.1038/s44303-026-00155-w

**Published:** 2026-04-08

**Authors:** Sandra Deiser, Sebastian Fenzl, Victor König, Shigeyoshi Inoue, Angela Casini

**Affiliations:** 1 Chair of Medicinal and Bioinorganic Chemistry, Department of Chemistry, School of Natural Sciences, Technical University of Munich, München, 85748 Germany; 2 Chair of Pharmaceutical Radiochemistry, Department of Chemistry, School of Natural Sciences, Technical University of Munich, München, 85748 Germany; 3 Chair of Silicon Chemistry, Department of Chemistry, School of Natural Sciences, Technical University of Munich, München, 85748 Germany

**Keywords:** Cancer, Chemistry, Drug discovery, Oncology

## Abstract

The radiohybrid (rh) design of radiopharmaceuticals has recently produced new theranostics suitable for both positron emission tomography (PET) imaging and peptide receptor radionuclide therapy (PRRT). This approach aims to address the limitations of current medical radionuclides by offering a new strategy for combining radionuclides that previously lacked both therapeutic and diagnostic applications. Here, we report on a somatostatin receptor subtype 2 (sstR2)-targeted radiohybrid compound, rhTATE4, which features a bifunctional silicon-based fluoride acceptor (SiFA) - named (SiFA)*SeFe* - for ^18^F-labeling, along with a DOTA (1,4,7,10-tetraazacyclododecane-1,4,7,10-tetraacetic acid) chelator for ^177^Lu-coordination. The rh-theranostic agent demonstrates similar in vitro behavior compared to the gold standards [^177^Lu]Lu-DOTA-TATE and SiFA*lin*-TATE, along with an exceptionally high tumor uptake (53.58 ± 5.51% ID/g for the radiofluorinated version) after 1 h post-injection in AR42J tumor-bearing mice, making it ideal for imaging. Moreover, clearance from normal tissues and considerable tumor retention (10.32 ± 7.04%ID/g) for [^177^Lu]Lu-TATE4 were observed at 24 p.i., suggesting good therapeutic applicability.

## Introduction

The term “theranostics” refers to the integration of two medical procedures that enable both diagnostic imaging and therapeutic treatment. In the context of nuclear medicine, this involves the use of identical or similar radiopharmaceutical compounds that contain radioactive isotopes^1–3^. A classical categorization includes the following main strategies for theranostic design: isotopically matched pairs, the use of “true” theranostic nuclides, and matched radiopharmaceuticals.

The iodine radioisotopes serve as a notable example of *isotopically matched pairs*, being the first radiopharmaceutical that laid the groundwork for theranostic applications^4,5^. Thus, iodine-123 (*γ*-emitter) is used for diagnostics in single-photon emission computed tomography (SPECT), and iodine-131 (*γ*- and *β*^−^-emitter) for the treatment of thyroid cancer^6^. The radiopharmaceuticals of this group contain either a therapeutic or a diagnostic radionuclide of the same element, whereby both compounds behave chemically and biologically identically, enabling precise dosimetry and therapy control^7^.

In the case of a “*true”theranostic* approach, the incorporated radionuclide emits either *γ*-rays or positrons that can be used for imaging, and *α* or *β*^−^ radiations for therapy. The first nuclide of this category is the aforementioned I-131 for imaging and therapy of thyroid cancer^8^. Another prominent example of this approach is the nuclide lutetium-177, which is already used in the clinic within the high-affinity somatostatin receptor subtype 2 (sstR2)-targeted [^177^Lu]Lu-DOTA-TATE (TATE = (Tyr^3^)-octreotate, DOTA = 1,4,7,10-tetraazacyclododecane-1,4,7,10-tetraacetic acid) (Lutathera^®^) for the therapy of neuroendocrine tumors^9,10^. The *γ*-rays from lutetium-177 can be utilized for SPECT imaging, allowing dosimetry calculations to be performed after the first therapy cycle^11^. However, ^177^Lu-based SPECT scans are not used in clinical practice for therapy planning due to their lower resolution^12^. To overcome this limitation, the *matched radiopharmaceuticals* approach provides a combination of imaging and therapeutic nuclides. In the case of neuroendocrine tumors, [^68^Ga]Ga-DOTA-TATE (NETSPOT^®^) is used for positron emission tomography (PET) imaging as complementary to the therapeutic [^177^Lu]Lu-DOTA-TATE^13–16^. However, the differing radiometal complexes of Ga^3+^ and Lu^3+^ result in distinct pharmacokinetic profiles, leading to diverse pharmacological properties^17–22^.

**Fig. 1:**
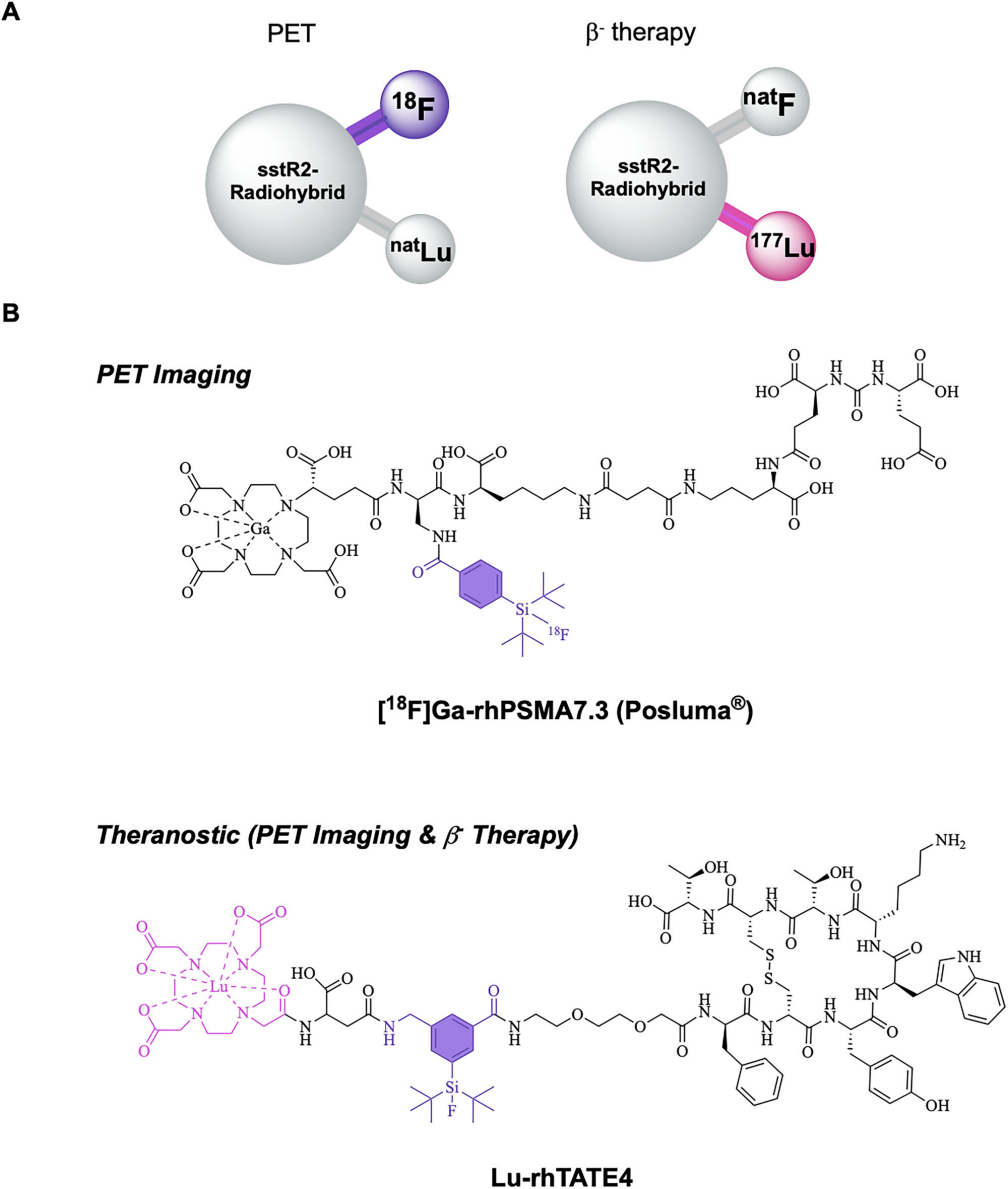
Radiohybrid tracer design and examples. **A** Schematic representation of the radiohybrid (rh) strategy targeted to sstR2 (somatostatine receptor subtype 2). The rh design enables labelling the same compound with either fluorine-18 for diagnostic imaging (purple) or with lutetium-177 for radioligand therapy (magenta). Typically, a SiFA synthon is used for ^18^F-fluorination, while a chelator is applied for radiometalation (e.g., with Lutedium-177). **B** Structure of the FDA-approved [^18^F]Ga-rhPSMA-7.3 (POSLUMA^®^) for PET imaging of prostate cancer. C) Structure of the herewith reported sstR2-targeted rh-compound **Lu-rhTATE4** for both PET imaging and β^−^ therapy.

In addition to the aforementioned classical design approaches, the *radiohybrid* strategy is emerging, whereby a single molecule features two separate binding sites for different radionuclides (Fig. 1A)^23–25^. This aims to establish a more consistent pharmacological profile for the radioconjugate, resulting in more personalized and effective treatment plans. In detail, as shown in Fig. 1A, the concept typically includes a silicon-based fluoride acceptor (SiFA) for fast and efficient ^18^F-fluorination *via* an ^18^F/^19^F-isotope exchange reaction, as well as the chelator DOTA (2-[1,4,7,10-Tetraazacyclododecane]-pentanedioic acid) for radiometalation (e.g., with ^177^Lu)^24^. This creates a chemically identical compound pair (either ^nat^F/radiometal or F-18/non-radioactive metal) that enables precise dosimetry and therapy monitoring. Moreover, it provides economic and strategic advantages, as the approval for the therapeutic radiohybrid can be streamlined following the approval of the diagnostic counterpart^26,27^. The first example of this approach was reported by Wurzer et al.^27^, and successfully reached FDA approval with the compound [^18^F]Ga-rhPSMA-7.3 (POSLUMA^®^, Fig. 1B) for PET imaging of prostate cancer^28^. The therapeutic counterpart [^177^Lu]Lu-rhPSMA7.3 and its DOTA-analogue [^177^Lu]Lu-rhPSMA10.1 showed promising preclinical results^29,30^.

A primary concern in the classical design of radiohybrid tracers is the high lipophilicity of the SiFA building block (SiFA)BA (4-(di-tert-butylfluorosilyl)benzoic acid), which affects the biodistribution of the resulting radiotracers and may lead to unfavorable in vivo performance^31–33^. In a recent attempt to generate an sstR2-targeted radiohybrid compound, based on the more hydrophilic yet *para*-functionalized SiFA*lin* moiety, Wendlinger et al. could not exceed the performance of the benchmark SiFA*lin*-TATE in biodistribution studies in terms of tumor accumulation, although featuring a markedly reduced liver uptake at 1 h p.i. (post injection)^34^. To address this limitation, we have recently reported on a new bifunctional SiFA synthon 3-amino-5-(di-tert-butylfluorosilyl)benzoic acid ((SiFA)*SeFe*), that includes a carboxylic acid and an amine group located at the two meta-positions relative to the Si moiety^35^. This feature not only reduces the lipophilic nature of the SiFA synthon but also enables its bridged incorporation into a radiotracer, akin to a classical amino acid. (SiFA)*SeFe* has been recently used to develop three different rh-compounds (**rhTATE1-3**) that showed high accumulation in AR42J tumor-bearing female CD1-nu/nu mice (up to 27%ID/g 1 h p.i.), but also in the kidneys (up to 99%ID/g 1 h p.i.)^35^. Although total tumor uptake was increased compared to [^18^F]SiFA*lin*-TATE (18.51 ± 4.89%ID/g 1 h p.i.), the tumor-to-background (T/B) ratios for most organs were comparable or lower. Additionally, the rapid renal excretion of [^18^F]Lu-rhTATE3 likely prevents its therapeutic application.

Here, we report on an optimized theranostic radiohybrid ligand **rhTATE4**, which can be quickly and efficiently labeled with fluorine-18 using the (SiFA)*SeFe* moiety, as well as stably labeled with lutetium-177 via a DOTA chelator (Fig. 1B). In this instance, the bifunctional (SiFA)*SeFe* is incorporated bridging the sstR2 targeting TATE and the chelator. The compound was characterized by RP-HPLC (reverse-phase high-performance liquid chromatography) and HR-ESI-MS (high-resolution electrospray ionization mass spectrometry). Further, the in vitro properties of the ligand, including lipophilicity, human serum albumin (HSA) binding, sstR2 receptor binding affinity, as well as stability in human plasma, were evaluated and compared to the FDA and EMA approved benchmark for PRRT [^nat/177^Lu]Lu-DOTA-TATE, as well as to the clinically tested PET tracer [^nat/18^F]SiFA*lin*-TATE^36^. Additionally, the biodistribution of **[**^**18**^**F]Lu-rhTATE4** was investigated in vivo in AR42J tumor-bearing CD1-nu/nu mice at 1 h p.i. Similarly, **rhTATE4** was labeled with lutetium-177 and its biodistribution studied at 1 h, 6 h, 24 h p.i. in the same model.

## Results

### Design and synthesis

To generate a theranostic platform, the rh-concept was applied to the TATE-based tracer **rhTATE4**, featuring the SiFA building block (SiFA)*SeFe* for ^18^F-labeling and a terminal DOTA chelator for ^177^Lu-labeling (Fig. 1B). Unlike previously reported rhTATE1-3 tracers^35^, the design concept uses (SiFA)*SeFe* to bridge TATE to DOTA rather than leaving it in a terminal position. This, in principle, enables better shielding of the lipophilic SiFA group by hydrophilic moieties and, at the same time, guarantees the DOTA heptacoordination to lutetium-177 for therapy. To maintain a good affinity to sstR2, the linker unit 8-amino-3,6-dioxaoctanoic acid was first placed between TATE and the SiFA synthon, analogous to SiFA*lin*-TATE. Moreover, a d-Asp linker was introduced between the DOTA chelator and the SiFA moiety, since the carried negative charge was advantageous for stability in previous work on model amino acid constructs^35^.

The metal-free ligand and the corresponding references were synthesized by fluorenylmethoxycarbonyl standard peptide synthesis on solid phase (Fmoc SPPS) using a 2-chlorotrityl chloride (2-CTC) resin (see SI for details, Table S1), yielding 3 – 19% RP-HPLC purified precursors (chemical purity >95%, determined by RP-HPLC and mass spectrometry, Figs. S1–S10). Non-radioactive lutetium labeling was performed quantitatively with a 2.5-fold excess of LuCl_3_ at 80 °C for 15 min (Figs. S4 and S9). The solution was used with the excess LuCl_3_ without further purification.

### Radiolabelling

The ^18^F-labeling of the SiFA moiety was carried out manually according to a procedure described in the literature^37^. The radiofluorination of the tracers was completed within 30 min, resulting in radiochemical yields (RCY) > 38% after a short cartridge purification (Oasis^®^ HLB plus light cartridge, 30 µm particle size), and radiochemical purities (RCP) > 98%. The confirmation of peptide integrity and the quality controls are shown in the supplementary materials (Figs. S11–S19, and Table S3). Radiolabelling of **rhTATE4** with lutetium-177 was achieved within 5 min at 70 °C with RCY and RCP > 97% (radio-RP-HPLC and radio-TLC, Figs. S14 and S19) and molar activities of 30 GBq/µmol. After radiolabelling, all peptides were used without further purification.

### In vitro evaluation

**Fig. 2:**
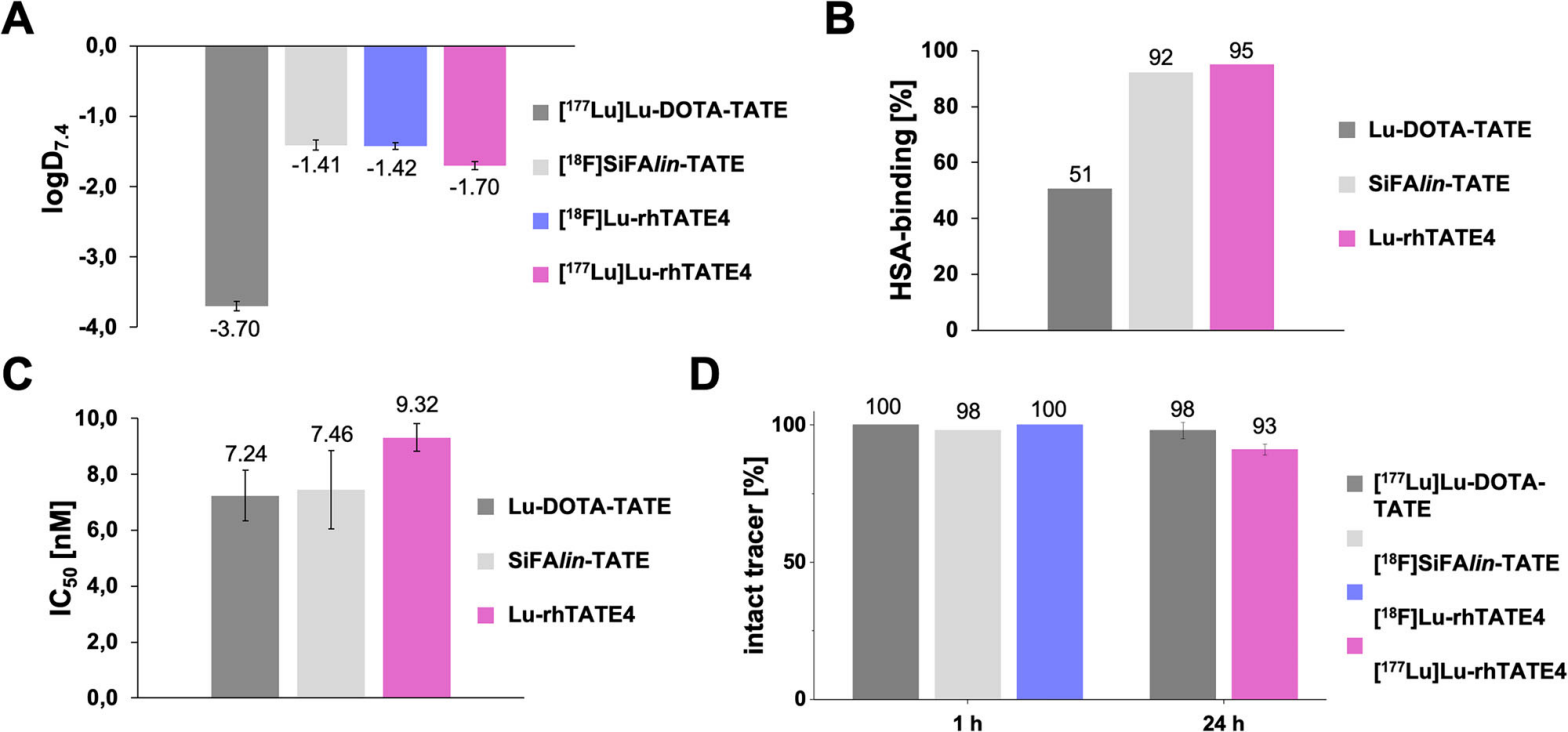
In vitro evaluation of the rh tracer. Determination of **A** lipophilicity (logD_pH=7.4_); **B** human serum albumin (HSA) binding (HPAC method, *n* = 2); **C** sstR2 binding affinity (IC_50_) in a competitive binding assay with the competitor [^125^I]I-TOC in the AR42J cell line; and **D** human plasma stability of the ^18^F- or ^177^Lu-labelled theranostic radiohybrid compound **rhTATE4** in comparison to benchmarks [^nat/177^Lu]Lu-DOTA-TATE and [^nat/18^F]SiFAlin-TATE. The value for the stability of [^18^F]SiFAlin-TATE was taken from the literature^40^.

The compound **rhTATE4** was evaluated in vitro and compared with the clinical standards [^nat/177^Lu]Lu-DOTA-TATE and [^nat/18^F]SiFA*lin*-TATE, including determination of lipophilicity, HSA binding, receptor binding affinity to sstR2-expressing CHO_sstR2_ cells, and human plasma stability (see SI for details, Table 1). The lipophilicity was determined using the 1-octanol-PBS partition coefficient at pH = 7.4 (log*D*_pH=7.4_) by the shake flask method. Both ^18^F- and ^177^Lu-labeled **rhTATE4** were investigated to estimate their chemical equality. Minor differences were observed for the ^18^F- and ^177^Lu-labeled species with log*D*_pH = 7.4_: −1.42 ± 0.05 and −1.70 ± 0.06, respectively. These differences are likely attributed to the different labeling conditions (see “Experimental” section for details). Overall, these log*D*_pH=7.4_ values were comparable to those of the previously reported rhTATE1-3^35^ and of SiFA*lin*-TATE (Fig. 2A); while the reference [^177^Lu]Lu-DOTA-TATE was the most hydrophilic compound (log*D*_pH=7.4_: −3.70 ± 0.05).

**Table 1:** Summary of in vitro data, including lipophilicity (logD_ph7.4_, *n* = 6), human serum albumin binding (HSA, *n* = 1), binding affinity (IC_50_, *n* = 3), and stability studies in human serum (% intact tracer, *n* = 3) after 1 h (^18^F-labelled, except for [^177^Lu]Lu-DOTA-TATE) and 24 h (^177^Lu-labelled) incubation at 37 °C

Compound	log*D*_ph=7.4_		HSA [%]	*IC*_50_ [nM]	Intact tracer [%]	
	^18^F	^177^Lu			1 h	24 h
[^nat/177^Lu]Lu-DOTA-TATE	–	−3.70 ± 0.05	51	7.24 ± 0.91	≥99 ± 0.0	98 ± 3.0
[^nat/18^F]SiFA*lin*-TATE	−1.41 ± 0.07	–	92	7.46 ± 1.40	98 ± 0.0	–
**[**^**nat/18**^**F][**^**nat/177**^**Lu]-rhTATE4**	${-}$1.42 ± 0.05	−1.70 ± 0.06	95	9.32 ± 0.49	≥99 ± 0.0	91 ± 2.0

The binding to HSA was determined using high-performance affinity chromatography (HPAC, Fig. S20, and Table S2). **Lu-rhTATE4** showed high binding to HSA of >92%, comparable to SiFA*lin*-TATE (Fig. 2B). Instead, markedly reduced HSA binding was observed for the more hydrophilic Lu-DOTA-TATE (51%)^38,39^. To evaluate the binding affinity to sstR2, the half-maximal inhibitory concentration (*IC*_50_) of **Lu-rhTATE4** was examined in a competitive binding assay ([^125^I]I-TOC as competitor) using CHO_sstR2_ cells (Chinese hamster ovary (CHO) cells stably transfected with human sstR2 (epitope-tagged at the *N*-terminal side)) (Fig. 2C). Compared with the benchmark compounds Lu-DOTA-TATE (*IC*_*50*_ = 7.24 ± 0.9 nM) and SiFA*lin*-TATE (*IC*_*50*_ = 7.46 ± 1.40 nM), **Lu-rhTATE4** demonstrated a similar receptor-binding affinity, with an *IC*_*50*_ of 9.32 ± 0.49 nM. This similarity suggests that the linker sequence 8-amino-3,6-dioxaoctanoic acid used in **Lu-rhTATE4** effectively retains receptor affinity.

Stability studies in human plasma were conducted by incubating the ligands for 1 h (^18^F-labeled) and 24 h (^177^Lu-labeled) at 37 °C (Fig. 2D). **[**^**18**^**F]Lu-rhTATE4**, as well as the reference [^18^F]SiFA*lin*-TATE^40^, showed no degradation after 1 h incubation in human serum (≥98% intact tracer). This confirms the stable fluorination of the bridged (SiFA)*SeFe* building block. With regard to therapeutic applications, the reference ligand [^177^Lu]Lu-DOTA-TATE and the radiohybrid **[**^**177**^**Lu]Lu-rhTATE4** also revealed a high stability after 24 h incubation in human plasma (>90% intact tracer).

### Ex vivo biodistribution

**Fig. 3:**
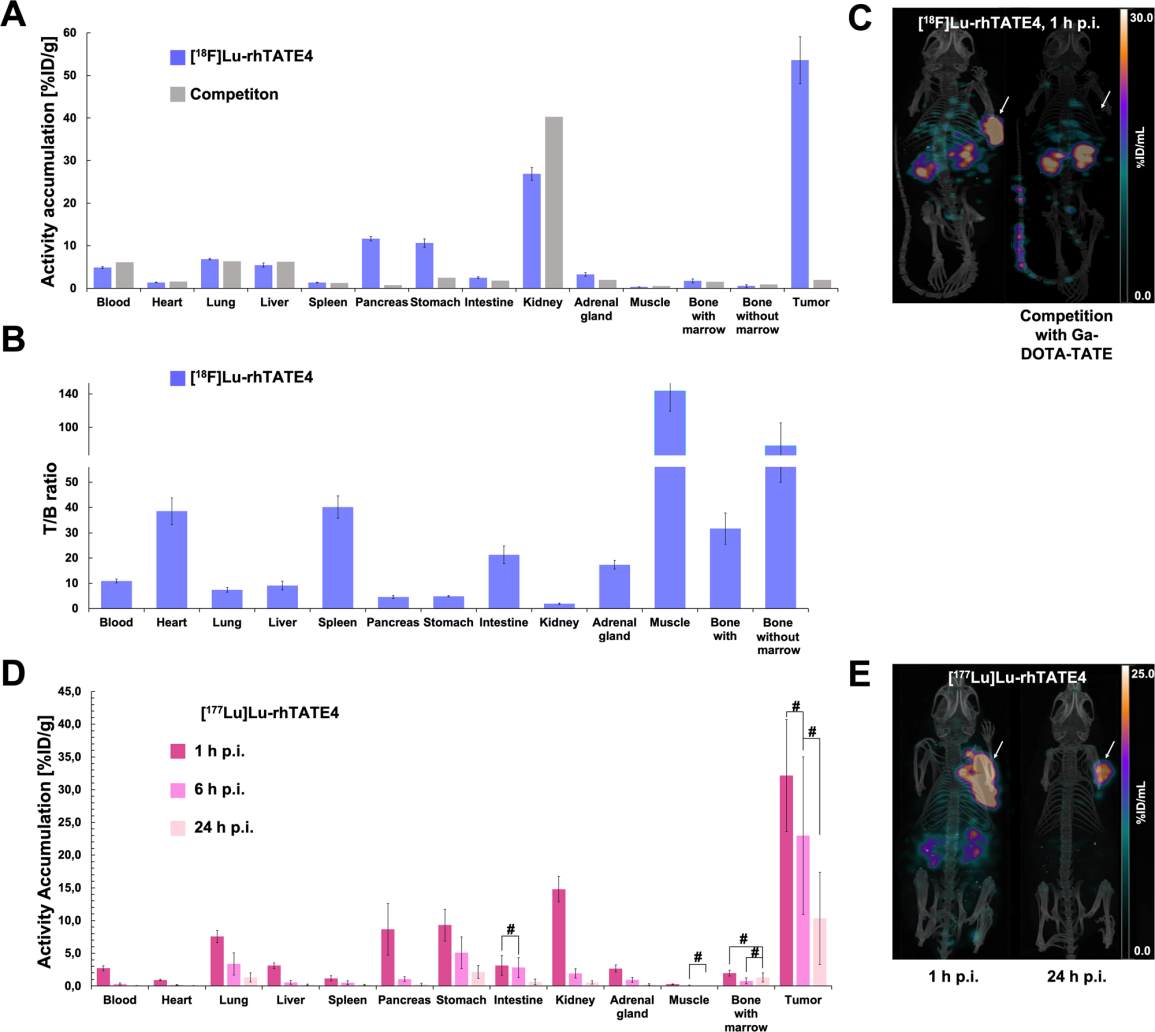
Ex vivo biodistributions and µSPECT/CT scans performed in AR42J tumor-bearing female CD1 nu/nu mice. **A** Ex vivo biodistribution data of **[**^**18**^**F]Lu-rhTATE4** (*n* = 3, 300 pmol each) and competition study (47 pmol + 40 nmol Ga-DOTA-TATE, *n* = 1) in selected organs 1 h post injection (p.i.) (exact values Table S5). **B** Tumor to background (T/B) ratios of **[**^**18**^**F]Lu-rhTATE4** in selected organs after 1 h p.i (300 pmol each). Hash mark indicates non-statistically significant differences (*p* > 0.05) (see Table S6 for complete analysis). **C** Maximum intensity projections (MIPs) of *µ*SPECT/CT scans acquired 1 h p.i. of **[**^**18**^**F]Lu-rhTATE4** (left, 40 pmol) and competition study (right, co-injection of 47 pmol **[**^**18**^**F]Lu-rhTATE4** + 40 nmol Ga-DOTA-TATE, *n* = 1). Tumors are indicated with white arrows. **D** Ex vivo biodistribution data of **[**^**177**^**Lu]Lu-rhTATE4** in selected organs at 1 h, 6 h and 24 h p.i. (*n* = 5, 300 pmol each). **E** MIPs of *µ*SPECT/CT scans acquired 1 h p.i. (left) and 24 h p.i. (right) of **[**^**177**^**Lu]Lu-rhTATE4** (300 pmol each). Tumors are indicated with white arrows. Static *µ*SPECT/CT images in **C** and **E** were acquired post mortem (CO_2_ asphyxiation and cervical dislocation) and after cardiac puncture with an acquisition time of 45 min.

To evaluate the imaging capabilities of **rhTATE4**, the ^18^F-labeled compound was first analyzed in AR42J tumor-bearing CD1 nu/nu mice after 1 h post injection (p.i.). Additional competition studies with co-injection of Ga-DOTA-TATE (40 nmol, 851x excess with respect to **[**^**18**^**F]Lu-rhTATE4**) were carried out in one mouse to determine the specificity to sstR2 (Fig. 3A, see SI for details, Table S4). After 1 h p.i., extremely high activity levels of 53.58 ± 5.51%ID/g were detected for **[**^**18**^**F]Lu-rhTATE4** in the AR42J tumor, while uptake in non-target tissues (heart, spleen, intestine, muscle, bone with and without bone marrow: 0.34–2.50%ID/g) was low. A slightly increased accumulation of activity was observed in the blood (4.88 ± 0.23%ID/g) and in the well-perfused lung (6.87 ± 0.21%ID/g), as well as in the liver (5.47 ± 0.47%ID/g). In addition, increased activity levels were observed in the pancreas (11.65 ± 0.51%ID/g) and stomach (10.63 ± 0.99%ID/g), which was expected due to endogenous sstR2 expression in these organs^41^. The observed accumulation in the kidneys (26.88 ± 1.49%ID/g) indicates primarily renal excretion.

In a competition study (*n* = 1) with co-injected excess Ga-DOTA-TATE, a sstR2-specific uptake of **[**^**18**^**F]Lu-rhTATE4** was verified in the tumor (1.96%ID/g), pancreas (0.75%ID/g) and stomach (2.50%ID/g). In contrast, no reduction in uptake was noted in the lungs, intestine, and adrenal glands, suggesting that the binding in these areas is primarily non-sstR2-mediated. The comparatively high accumulation of renal activity during the competition experiment can be attributed to receptor saturation by the cold competitor, resulting in an increased excretion rate of the radioligand. For a more precise assessment of the imaging quality, the T/B ratios were determined (Fig. 3B, for more details see SI, Table S5). **[**^**18**^**F]Lu -rhTATE4** exhibits high T/B ratios for the lung (7.37 ± 0.97), the spleen (40.13 ± 4.45), the pancreas (4.54 ± 0.54), intestine (21.31 ± 3.54), adrenal glands (17.33 ± 1.73) and the bones with (31.61 ± 6.12) and without marrow (77.5 ± 27.54). Only for kidneys (1.85 ± 0.26) and blood (10.87 ± 0.78), lower T/B ratios were reached.

One mouse of each biodistribution study was chosen for representative *µ*SPECT/CT scans of **[**^**18**^**F]Lu-rhTATE4** and the competition study of **[**^**18**^**F]Lu-rhTATE4** with Ga-DOTA-TATE after euthanization. The resulting *µ*SPECT/CT images recorded 1 h p.i. (45 min acquisition time) are shown in Fig. 3C and are in agreement with the ex vivo biodistribution studies, displaying favorable imaging characteristics for **[**^**18**^**F]Lu-rhTATE4**, with high activity uptake in the sstR2-overexpressing tumor. Furthermore, low accumulation in non-target tissues and preferential renal excretion were observed. Since no bone uptake could be observed in both images, the in vivo stability against defluorination was further confirmed.

In order to investigate long-term in vivo behavior of the compound, the ^177^Lu-labeled **rhTATE4** was evaluated at 1, 6, and 24 h p.i. in AR42J tumor-bearing CD1 nu/nu mice (Fig. 3D, and Tables S4 and S6). The biodistribution data of **[**^**177**^**Lu]Lu-rhTATE4** are in line with those of **[**^**18**^**F]Lu-rhTATE4** at 1 h p.i. indicating comparable in vivo performance; although statistically different absolute values of ^18^F- and ^177^Lu-labeled **rhTATE4** are recorded in the kidney (26.88 ± 1.49%ID/g vs. 14.80 ± 1.95%ID/g, respectively) and in the tumor (53.58 ± 5.51%ID/g vs. 32.15 ± 8.55%ID/g, respectively) amongst others (Table S7). The reason for the observed discrepancies between the two tracers is not yet fully understood. In any case, **rhTATE4** tumor uptake exceeded that achieved with the previously reported theranostic [^18^F]Lu-rhTATE3 compound (27.32 ± 8.86%ID/g; see also Table S8 for statistical analysis), while excessive renal excretion was prevented^35^. Most importantly, the compound accumulates more in the tumor than what has been reported for [^177^Lu]Lu-DOTA-TATE (21.35 ± 5.90%ID/g); although a different mouse model was used to grow the AR42J tumor in this case^42^.

After 24 h p.i., a considerable amount of activity could still be observed in the tumor (10.32 ± 7.04%ID/g), which is only slightly lower than the therapeutic benchmark [^177^Lu]Lu-DOTA-TATE in an AR42J xenograft model (16.14 ± 2.07%ID/g 24 h p.i.)^42^. Of note, markedly lower off-target uptake of **[**^**177**^**Lu]Lu-rhTATE4** compared to [^177^Lu]Lu-DOTA-TATE was observed in certain organs (lung: 1.30 ± 0.68%ID/g vs 11.89%ID/g, pancreas: 0.26 ± 0.18%ID/g vs 3.99 ± 0.47%ID/g, adrenal gland: 0.22 ± 0.14%ID/g vs 4.70 ± 1.07%ID/g, kidney: 0.55 ± 0.26%ID/g vs. 1.76 ± 0.35%ID/g respectively), favorable for future therapeutic application of the new rh-compound. The T/B ratios of **[**^**177**^**Lu]Lu-rhTATE4** were directly compared over time (Fig. S21, and Table S6), showing a steady increase in all organs relative to the benchmark compound.

One mouse of each biodistribution study was chosen for representative *µ*SPECT/CT scans of **[**^**177**^**Lu]Lu-rhTATE4** after euthanization. Images were acquired at 1 h and 24 h p.i. (Fig. 3E). *µ*SPECT/CT images showed activity accumulation in the AR42J xenografts, and slightly elevated renal levels at 1 h p.i. (Fig. 3F, left), in line with what was observed for **[**^**18**^**F]Lu-rhTATE4**. In agreement with quantitative data from biodistribution studies, the activity was efficiently cleared through the kidneys over time and almost entirely excreted after 24 h (Fig. 3F, right). In addition, good excretion from non-cancerous tissues was also confirmed after 24 h p.i.

## Discussion

Molecular theranostic approaches have gained significant importance in modern medicine over the past years. To date, only a limited number of radiohybrid compounds have been identified as next-generation theranostic agents, in which the PET nuclide F-18 is primarily introduced through an isotopic exchange reaction via the highly lipophilic silicon-based fluoride acceptor (SiFA) moiety^24^. Different strategies have been employed to enhance the radiohybrid hydrophilicity, including the introduction of polar auxiliaries, as seen in SiFA*lin*-TATE, or the use of trifluoroborate-based radioprosthetic for ^18^F-exchange^23,43^. In our work, the rh-compound **rhTATE4** features the bifunctional synthon (SiFA)*SeFe*, bridging the DOTA chelator to the TATE peptide. Notably, the compound exhibited reduced lipophilicity with respect to previously developed SiFA-based radiohybrids, and was comparable to that of the optimized [^18^F]SiFA*lin*-TATE^35^. The labeling of **rhTATE4** with fluorine-18 and lutetium-177 could be efficiently implemented, with high RCYs and RCPs. The derivative **[**^**nat/18**^**F][**^**nat/177**^**Lu]Lu-rhTATE4** showed high HSA binding and stability in serum, as well as comparable sstR2 binding affinity to the benchmark Lu-DOTA-TATE. Initial ex vivo evaluation of **[**^**18**^**F]Lu-rhTATE4** demonstrated noticeable imaging properties at 1 h p.i.; moreover, receptor specificity was confirmed by the lack of uptake in the tumor and other sstR2-positive organs in competition experiments. Furthermore, biodistribution studies of **[**^**177**^**Lu]Lu-rhTATE4** revealed high tumor accumulation even at 24 h p.i., comparable to the benchmark [^177^Lu]Lu-DOTA-TATE.

Overall, our study further demonstrates the potential of the radiohybrid approach for the development of targeted theranostics. Additionally, it highlights the advantages of the (SiFA)*SeFe* moiety, enabling a more versatile design. Further work will be devoted to incorporating this synthon into rh-tracers targeting different receptors, such as the chemokine receptor 4 (CXCR4) or the gastrin-releasing peptide (GRPR) receptor, to broaden the scope of peptide-based theranostics^44^.

## Methods

### General

The solvents were obtained from technical or in HPLC-grade from *Sigma-Aldrich (Munich, Germany*), *Alfa Aesar* (Karlsruhe, Germany), *Carl Roth GmbH & Co. KG* (Karlsruhe, Germany) or *VWR International GmbH* (Darmstadt, Germany). The water for the HPLC solvents was supplied by the in-house Millipore *Barnsted MicroPure-*System from *Thermo Fischer Scientific Inc*. (Morrisville, PA, USA). The *Ultrapur*^*®*^ water and DMSO (anhydrous) were purchased from *Sigma Aldrich* (Munich, Germany). Synthesis of *(SiFA)SeFe* was performed according to established procedures^35^. Chemicals for liquid phase synthesis and deuterated solvents were obtained from *VWR International GmbH* (Darmstadt, Germany), *Carl Roth GmbH & Co. KG* (Karlsruhe, Germany, *Tokyo Chemical Industry* (Tokyo, Japan)*, Gelest Inc*. (Morrisville, PA, USA), and *Sigma-Aldrich (Munich, Germany*) and used without further purification. Amino acids were sourced from *Carbolution Chemicals GmbH* (St. Ingbert, Germany), *IRIS Biotech GmbH* (Marktredewitz, Germany), *Millipore-Sigma HGaA* (Darmstadt, Germany) or *Sigma Aldrich* (Munich, Germany). The Chelator DOTA(*t*Bu)_3_ was obtained from *CheMatech* (Dijon, France). Coupling reagents as well as other chemicals required for peptide synthesis were acquired from *Macrocyclics Inc*. (Dallas, USA), *Molekula GmbH* (Garching, Germany) or *Sigma Aldrich* (Munich, Germany). The 2-CTC-resin was purchased from *IRIS Biotech GmbH* (Marktredewitz, Germany). Thallium(III) trifluoroacetate, glycerol, imidazole, and hydroxylammonium chloride were purchased from *Sigma Aldrich* (Munich, Germany). All reagents were used without further purification. Cell culture media DMEM/F-12 (1:1) + GlutaMAX™-I and RPMI medium 1640 were purchased from *Thermo Fisher Scientific* (Waltham, Massachusetts, USA). Media additives such as fetal bovine serum (FBS), l-Gln solution (200 mM), and MEM non-essential amino acid solution were acquired from *Bio&SELL* (Nürnberg, Germany) and *Sigma Aldrich* (Munich, Germany), respectively. Hanks’ balanced salt solution (HBSS), Phosphate-Buffered Saline pH = 7.4 (PBS) and Trypsin/EDTA (0.05%/0.02% in PBS without Ca^2+^/Mg^2+^) were purchased from *Sigma Aldrich* (Munich, Germany). Bovine serum albumin (BSA) and Trypan Blue (0.4%) were purchased from *Biowest* (Nuaillé, France).

Radioactive labeling with Iodine-125 for affinity studies was performed with a [^125^I]NaI-solution in 40 mM NaOH (74 TBq/mM) from *HARTMANN ANALYTIC GmbH* (Braunschweig, Germany). Iodogen for the synthesis was purchased from *Pierce™ Biotechnology* (Rockford, IL, United States). Radioactive labeling solution with fluorine-18 in target water ([^18^O]H_2_O) was supplied by *Klinikum rechts der Isar* (Munich, Germany). For ^177^Lu-labeling, a solution of [^177^Lu]LuCl_3_ (1 GBq) Radiochemical Grade EndolucinBeta^®^ n.c.a. (40 GBq/mL) in 0.04 M HCl was acquired from *ITM Isotope Technologies Munich SE* (Munich, Germany). The Cartridges for ^18^F-labeling were supplied by *Waters GmbH* (Eschborn, Germany). Protein LoBind^®^ tubes were purchased from *Eppendorf SE* (Hamburg, Germany). Silica gel 60 coated aluminum stripes and iTLC-SG stripes, and for reaction controls were obtained from *Agilent* (Santa Clara, California, USA) and *Merck KGaA* (Darmstadt, Germany), respectively. The 24-well plates for *IC*_50_ studies were supplied from *Thermo Scientific Inc*. (Waltham, Massachusetts, USA), and sterile cell culture flasks were acquired from *Greiner Bio-One GmbH* (Frickenhausen, Germany). Centrifugal filters for stability studies in human serum were purchased from *VWR International GmbH* (Darmstadt, Germany).

Analytical and preparative RP-HPLC was carried out on *Shimadzu Corp*. Instruments (Kyoto, Japan) equipped with two LC-20AD gradient pumps, a CBM-20A communications module and a Smartline UV detector 2500 (*λ* = 220 nm, λ = 254 nm) from *Dr. Ing. Herbert Knauer GmbH* (Berlin, Germany). For analytical RP-HPLC, a flow rate of 1.0 mL/min was used. Quality controls of peptic ligands were performed on a MultoKrom^®^ 100-5 C18-column (125 × 4.6 mm, 5 μm particle size, *CS Chromatographie GmbH*). Different gradients of MeCN (*J. T. Baker*^*®*^ ultra-gradient HPLC rate, mixed with 5% H_2_O and 0.1% TFA) in H_2_O (0.1% TFA) were used as eluents for all RP-HPLC operations. Preparative HPLC was performed with a Multospher® 100 C18-column (150 × 10 mm, 5 μm particle size, *CS Chromatographie GmbH*) at a flow rate of 5.0 mL/min. Different gradients of MeCN (*J. T. Baker®* ultra-gradient HPLC rate, mixed with 5% H_2_O and 0.1% TFA) in H_2_O (0.1% TFA) were used as eluents for all RP-HPLC operations. All RP-HPLC chromatograms were analyzed *via* the *LabSolutions* software.

Radiolabelled compounds were investigated by analytical Radio-RP-HPLC from *Shimadzu Corp*. (Kyoto, Japan), comprising of two LC-20AD gradient pumps, a DGU-20A degassing unit, a SIL-20A autosampler, a CTO-10AS column oven, a FRC-10A fraction collector, a SPD-20A UV/Vis detector (*λ* = 220 nm), a HERM LB500 (NaI-scintillation crystal) radio-detector (from the company *Berthold Technologies GmbH*, Bad Wilbad, Germany) and a CBM-20A communications module. For the characterization of radioactively labeled peptidic compounds, a Multospher® 100 C18-column (125 × 4.6 mm, 5 μm particle size, *CS Chromatographie GmbH*) was used.

Radioactive TLCs were analyzed using a Scan-RAM™ from *LabLogic* (Koblenz, Germany). The acquired data were processed using the Laura Software (Koblenz, Germany). For the quantification of the activities of radio-labeled compounds an automatic 2480 WIZARD2 γ counter from *PerkinElmer Inc*. (Waltham, USA) was used. For the quantification of some activities within ex vivo biodistribution studies with **[**^**177**^**Lu]Lu-rhTATE4**, an APEX y-counter with an GX4018 detector from *MIRION Technologies* (Atlanta, Georgia, USA) and a CP-5SL Cryo-Pulse^®^ 5 Cryostat from *CANBERRA* (Toledo, Spain) was used. All activities were analyzed *via* the Apex-Gamma software. The calibration curve was determined using Excel software (*Microsoft Corporation*, Redmond, WA, USA). ESI-MS spectra are recorded on an expression CMS mass spectrometer with a quadrupole analyzer and an electron spray ionizer (positive mode) (*Advion Inc*., Ithaca, New York, USA). All ESI-MS spectra were analyzed *via* the *Advion Mass Express* software. Freeze-drying was carried out on an Alpha 1.2 Lyophiliser from *Martin Christ Gefriertrocknungsanlagen GmbH* (Osterode im Harz, Germany) with a RZ-2 rotary vane pump from *Vacuubrand GmbH & Co. KG* (Wertheim, Germany). µSPECT/CT scans of mice were recorded on a VECTor4 small-animal SPECT/PET/OI/CT from *MILabs* (Utrecht, Netherlands). All scans were analyzed *via* the PMOD software from *PMOD Technologies LCC* (Fällanden, Switzerland).

### General synthesis methods (GS)

Solid-phase peptide synthesis using Fmoc strategy (Fmoc-SPPS): The resin is loaded with a Fmoc-protected amino acid at the *C*-terminus. The Fmoc protecting group is then removed and coupled to the next amino acid using coupling reagents. After completion, the peptide is cleaved from the resin.

GS1: Loading of the 2-CTC resin: The 2-chloro-tritylchlorid (2-CTC) resin (loading capacity: 1.6 mmol/g) was swollen for 30 min in 5 mL NMP and then washed with DMF (dimethylformamide, 6 × 5 mL). The resin was loaded with an Fmoc-protected amino acid (AA) using Fmoc-AA-OH (1.5 eq.) and DIPEA (*N*,*N*-Diisopropylethylamine) (1.5 eq.) in DMF (*N*,*N*-Dimethylformamide) in a 20 mL peptide syringe. After 15 min of pre-activation at room temperature (RT), another 3.0 eq. of DIPEA was added and the mixture was shaken at RT for 2 h. MeOH (1 mL/g resin) was added to the resin and shaken for 15 min. Afterwards, the resin was washed five times each with DMF (5 mL), MeOH (5 mL) and DCM (dichloromethane, 5 mL) and dried in a desiccator.

GS2: Standard peptide coupling to the resin: The loaded resin is swollen in NMP for 30 min, washed six times with DMF (5 mL), and *N*-terminally Fmoc deprotected. Prior to coupling at the *C*-terminus of side-chain-protected Fmoc-AA-OH (1.5 eq.), preactivation is performed with TBTU (1.5 eq.), HOAt (1.5 eq.), and DIPEA (4.0 eq.) in 5 mL DMF. After 10 min, the activated solution is added to the resin-bound peptide containing the free amine (2-CTC-AA-NH_2_) and shaken for 1.5 h at RT. The resin is then washed six times with DMF (5 mL) and, after Fmoc deprotection, washed another six times with DMF (5 mL). Now the next amino acid can be reacted, or washed six times with DCM and dried in a desiccator.

GS3: Deviating peptide coupling to the resin: After swelling the resin in NMP for 30 min and washing with DMF (6 × 5 mL), the respective substrate is coupled to the resin-bound peptide (Table S1). The resin is then washed six times with DMF (5 mL).

GS4: Fmoc deprotection: *N*-terminal Fmoc-protected amino acids or peptides bound to the resin are deprotected by adding 20% piperidine in DMF (5 mL). The deprotection reagent is added twice (1 × 5 min, 1 × 15 min). The resin is then washed with DMF (6 × 5 mL each).

GS5: Acetyl deprotection: Acetyl deprotection is performed by dissolving 50 µmol of the peptide in MeOH and adding NaOMe until the pH is 11–12. After 15 min, the reaction is stopped by adding TFA (pH = 2).

GS6: Cleavage from the resin with retention of acid labile protective groups: The peptide bound to the resin is mixed with 5 mL of 2,2,2-Trifluorethanol (TFE)/DCM/AcOH (3/6/1) and agitated for 20 min at RT. The solution with the protected peptide is collected and then evaporated under a nitrogen stream.

GS7: Cleavage from the resin with removal of acid-labile protection groups: The peptide bound to the resin is mixed with 5 mL of TFA/TIPS/H_2_O (95/2.5/2.5) and agitated twice for 45 min at RT. The solution with the deprotected peptide is collected in a 50 mL round-bottom flask, and the remaining resin is washed once with 5 mL TFA and stirred overnight. The following day, the TFA is evaporated under nitrogen stream.

GS8: Complexation with lutetium: For the incorporation of lutetium, LuCl_3_ (20 mM in H_2_O, 3.0 eq.) is added to a 2 mM solution of the compound in DMSO (dimethyl sulfoxide) and diluted to 1 mM by addition of DMSO. The obtained solution is incubated at 80 °C for 15 min.

GS9: Synthesis of the binding motif TATE on resin with protecting groups (PG) (H-TATE(PG)-2-CT): The synthesis of H-TATE(PG)-2-CT is carried out on the resin using the general working procedures (**GS**).
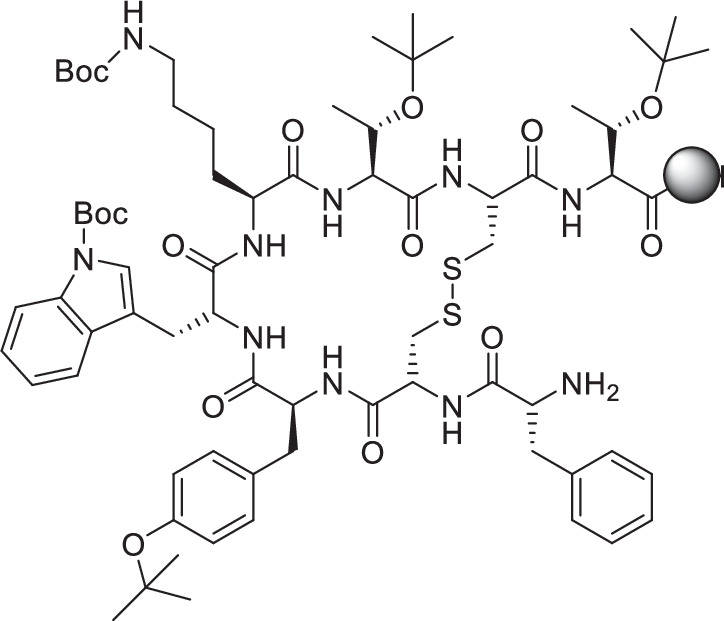


2-CTC resin is loaded with Fmoc-*L*-Thr(^*t*^Bu)-OH (**GS1**). This is followed by the coupling of Fmoc-*L*-Cys(Acm)-OH, Fmoc-*L*-Thr(^*t*^Bu)-OH, Fmoc-*L*-Lys(Boc)-OH, Fmoc-*D*-Trp(Boc)-OH, Fmoc-*L*-Tyr(tBu)-OH, Fmoc-*L*-Cys(Acm)-OH and Fmoc-*D*-Phe-OH (**GS2**). Before the coupling of the next amino acid in each case, the *N*-terminus is Fmoc-deprotected (**GS4**). The final amino acid is only deprotected after the formation of the disulfide bridge.

Formation of the disulfide bridge: Fmoc-*D*-Phe-*L*-Cys(Acm)-*L*-Tyr(tBu)-*D*-Trp(Boc)-*L*-Lys(Boc)-*L*-Thr(^*t*^Bu)-*L*-Cys(Acm)-*L*-Thr(^*t*^Bu)-2-CT (1.0 eq.) is mixed with Tl(TFA)_3_ (4.0 eq.) and glycerol (4.0 eq.) in DMF (8 mL/g resin). After 1 h at RT, the solution is discarded, and a fresh solution of the reaction solution is added to the resin for another 1 h at RT. The resin is then washed with DMF (6 × 5 mL/g resin). Test cleavage from the resin with retention of acid-labile protective groups is used to verify the completeness of the cyclization (**GS6**). Characterization is investigated by analytical RP-HPLC and ESI-MS. After final Fmoc deprotection, the product, H-*D*-Phe-cyclo[*l*-Cys-*L*-Tyr(^*t*^Bu)-*D*-Trp(Boc)-*L*-Lys(Boc)-*L*-Thr(^*t*^Bu)-*L*-Cys]-*L*-Thr(^*t*^Bu)-2-CT is present bound to the resin.

RP-HPLC (10-90% MeCN/H_2_O with 0.1% TFA, *v*/*v*, 15 min, *λ* = 220 nm) for H-TATE(PG)-OH: *t*_R_ = 13.4 min.

MS (ESI positive): *m*/*z* calculated for H-TATE(PG)-OH: 1416.7; found: 1418.3 [M + H^+^]^+^.

Synthesis of SiFA*lin*-TATE: The synthesis of SiFA*lin*-TATE is carried out on resin using the general working procedures (**GS**).
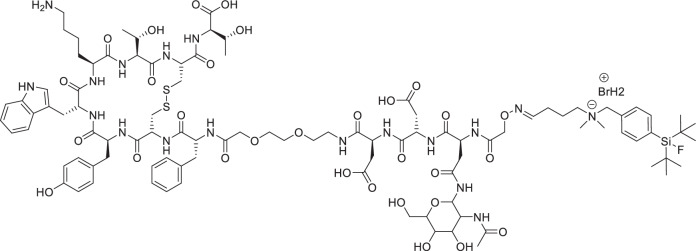


The resin-bound synthesis of H-TATE(PG)-2-CT (**GS9**) is followed by the coupling of as Fmoc-NH-((CH_2_)_2_O)_2_-COOH (**GS3**), Fmoc-*L*-Asp(O^*t*^Bu)-OH (**GS3**), Fmoc-*L*-Asp(O^*t*^Bu)-OH (**GS2**), Fmoc-Asn(Ac_3_AcNH-*β*-Glc)-OH (**GS3**) and *bis*-Boc-amino-oxyacetic acid (**GS3**). Before the coupling of the next amino acid in each case, the *N*-terminus is Fmoc-deprotected (**GS4**). After resin cleavage, removal of all protecting groups (**GS7**) and acetyl deprotection (**GS5**), purification is carried out by RP-HPLC (30–35% MeCN/H_2_O with 0.1% TFA, *v*/*v*, 20 min, *λ* = 220 nm).

Oximligation: 1.0 eq. TATE-O_2_OC-*L*-Asp-*L*-Asp-Asn-amino-oxy-acid and 4.0 eq. SiFA*lin* aldehyde in 400 µL phosphate buffer/MeCN (1/1, *v*/*v*) is basified with 4 N NaOH solution until a pH of pH = 4 is established. After 20 min, the solution is diluted 1/1 with H_2_O (+0.1% TFA) and purified by RP-HPLC (20-45-60% MeCN/H_2_O with 0.1% TFA, *v*/*v*, 10–30 min, *λ* = 220 nm), 3.41 mg (1.52 μmol, 5%) is obtained in the form of a white solid.

RP-HPLC (10–60% MeCN/H_2_O with 0.1% TFA, *v*/*v*, 15 min, *λ* = 220 nm) for SiFA*lin*-TATE: *t*_R_ = 13.5 min.

MS (ESI positive): *m*/*z* calculated for SiFA*lin*-TATE: 2241.9, found: 1082.5 [M + H^+^]^+^.

Synthesis of Lu-DOTA-TATE: The synthesis of Lu-DOTA-TATE is carried out on resin using the general working procedures (**GS**).
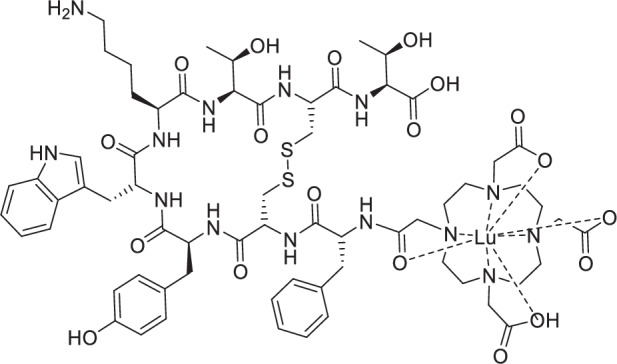


The resin-bound synthesis of H-TATE(PG)-2-CT (**GS9**) is followed by the coupling of DOTA(tBu)_3_ (**GS3**). After resin cleavage, removal of all protecting groups (**GS7**) and purification by RP-HPLC (15-40% MeCN/H_2_O with 0.1% TFA, *v*/*v*, 30 min, *λ* = 220 nm), 1.11 mg (7.73 μmol, 19%) DOTA-TATE is obtained in the form of a white solid. Afterwards, the incorporation of lutetium (**GS8**) was performed.

RP-HPLC (10–60% MeCN/H_2_O with 0.1% TFA, *v*/*v*, 15 min, *λ* = 220 nm) for Lu-DOTA-TATE: *t*_R_ = 8.5 min.

MS (ESI positive): *m*/*z* calculated for Lu-DOTA-TATE: 1606.5, found: 804.6 [M + 2H^+^]^2+^.

Synthesis of Lu-rhTATE4: The synthesis of **rhTATE4** is carried out on resin using the general working procedures (**GS**).
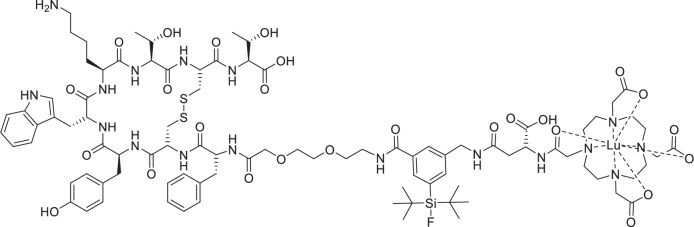


The resin-bound synthesis of H-TATE(PG)-2-CT (**GS9**) is followed by the coupling of Fmoc-NH-((CH_2_)_2_O)_2_-COOH (**GS3**), Fmoc-(SiFA)*SeFe*-OH (**GS3**), Fmoc-*D*-Asp-O^*t*^Bu (**GS3**) and DOTA(^*t*^Bu)_3_ (**GS3**). Before the coupling of the next amino acid in each case, the *N*-terminus is Fmoc-deprotected (**GS4**). After resin cleavage, removal of all protecting groups (**GS7**) and purification by RP-HPLC (30-50% MeCN/H_2_O with 0.1% TFA, *v*/*v*, 30 min, *λ* = 220 nm), 4.08 mg (2.05 μmol, 3%) is obtained in the form of a white solid. Afterwards, the incorporation of lutetium (**GS8**) takes place.

RP-HPLC (10–60% MeCN/H_2_O with 0.1% TFA, *v*/*v*, 15 min, *λ* = 220 nm) for Lu-rhTATE4: *t*_R_ = 13.0 min.

MS (ESI positive): *m*/*z* calculated for Lu-rhTATE4: 2159.8, found: 1081.0 [M + 2H^+^]^2+^.

### Radiolabelling

GS10: ^18^F-labeling: For ^18^F-labeling, the aq. fluorine-18 (120 MBq) is retained on a SAX cartridge (Sep-Pak Accel Plus QMA Carbonate Plus Light, 46 mg, Waters) preconditioned with 10 mL H_2_O. The cartridge is dried with air (10 mL), then washed slowly with dry DMSO (8 mL) and dried again with air (10 mL). The dried fluorine-18 is eluted with 500 µL ammonium formate (40.0 mg in 500 µL dry DMSO) into a 1.5 mL reaction tube (Protein LowBind, Eppendorf^®^). The reaction takes place by adding 30 µL (30 nmol) of the ligand (1 mM in DMSO) at 40 °C for 5 min. The reaction is stopped by adding 10 mL of PBS pH = 3 (adjusted with 1 M aq. HCl). The solution is then slowly loaded onto an Oasis HLB Plus Light cartridge (30 mg sorbent, 30 µm particle size) preconditioned with absolute EtOH (10 mL) and H_2_O (10 mL). The cartridge is washed with PBS pH = 7.4 (10 mL) and then dried with air (10 mL). The ^18^F-labeled ligand is eluted using an elution cocktail of abs. EtOH and PBS pH = 7.4 (7/3 v/v; 300 µL). Quality controls are performed by analytical radio-RP-HPLC (MeCN/H_2_O: 10–60% with 0.1% TFA in 15 min) and radio-thin layer chromatography (flow agent: 60% MeCN/ 40% PBS (6/4 v/v) with 10% NaOAc in H_2_O (2 M) and 1% TFA, stationary phase: TLC silica gel 60 F_254_ from Merck Millipore).

[^18^F]SiFAlin-TATE: ^18^F-labeling is carried out according to GS10. The formation of the desired product is confirmed by Radio-RP-HPLC and Radio-TLC.

Radio-RP-HPLC (MeCN/H_2_O: 10–60% with 0.1% TFA in 15 min): *t*_R_ = 15.5 min, RCY [d.c.] = 59%, RCP = 98%.

Radio-TLC (flow agent: 60% MeCN/ 40% PBS (6/4v/v) with 10% NaOAc in H_2_O (2 m) and 1% TFA, stationary phase: TLC silica gel 60 F_254_ from Merck Millipore): RCP = 98%.

[^18^F]Lu-rhTATE4: ^18^F-labeling is carried out according to GS10. The formation of the desired product is confirmed by Radio-RP-HPLC and Radio-TLC.

Radio-RP-HPLC (MeCN/H_2_O: 10–60% with 0.1% TFA in 15 min): *t*_R_ = 14.6 min, RCY [d.c.] = 38%, RCP = 99%.

Radio-TLC (flow agent: 60% MeCN/ 40% PBS (6/4v/v) with 10% NaOAc in H_2_O (2 m) and 1% TFA, stationary phase: TLC silica gel 60 F_254_ from Merck Millipore): RCP = 99%.

GS11: For ^177^Lu-labeling, the aq. [^177^Lu]LuCl_3_ (30 MBq) is added to 1 µL (1 nmol) of the ligand (1 mM stock in DMSO), 10 µL of a NaOAc buffer (pH = 5.5), 22 µL 0.04 M HCl and reacted at 70 °C for 5 min.

[^177^Lu]Lu-DOTA-TATE: ^177^Lu-labeling is carried out according to **GS11**. The formation of the desired product is confirmed by Radio-RP-HPLC and Radio-TLC.

Radio-RP-HPLC (MeCN/H2O: 10–60% with 0.1% TFA in 15 min): *t*_R_ = 8.5 min, RCY [d.c.] > 99%, RCP > 99%.

Radio-TLC (flow agent: 1 M NH_4_OAc/DMF (1/1, *v*/*v*), stationary phase: TLC silica gel 60 F_254_ from Merck Millipore): RCP > 99%.

Radio-TLC (flow agent: 0.1 M sodium citrate × 1.5 H_2_O, stationary phase: iTLC-SC from Merck Millipore): RCP > 99%.

[^177^Lu]Lu-rhTATE4: ^177^Lu-labeling is carried out according to **GS11**. The formation of the desired product is confirmed by Radio-RP-HPLC and Radio-TLC.

Radio-RP-HPLC (MeCN/H2O: 10–60% with 0.1% TFA in 15 min): *t*_R_ = 11.9 min, RCY [d.c.] = 99%, RCP = 99%.

Radio-TLC (flow agent: 1 M NH_4_OAc/DMF (1/1, *v*/*v*), stationary phase: TLC silica gel 60 F_254_ from Merck Millipore): RCP > 99%.

Radio-TLC (flow agent: 0.1 M sodium citrate × 1.5 H_2_O, stationary phase: iTLC-SC from Merck Millipore): RCP = 99%.

GS12: ^125^I-labeling to achieve [^125^I]I-TOC. For in vitro studies (*IC*_50_, *n* = 3), 50-150 µg of TOC are dissolved in 20 µL of DMSO and 280 µL of TRIS buffer (25 mM TRIS-HCl, 0.4 mM NaCl, pH = 7.5) in a 1.5 mL Eppendorf reaction tube (Protein LowBind). The solution is transferred to another reaction tube (1.5 mL, Protein LowBind) coated with Iodogen^®^ (150 µg) and 5.00 µL of (10–20 MBq) [^125^I]NaI (74 TBq, 40 mM NaOH, *HARTMANN ANALYTIC GmbH* (Braunschweig, Germany)) is added. After 15 min at RT, the reaction is stopped by separation from the oxidant (Iodogen^®^), and the crude product is purified by analytical RP-HPLC [(20–40% in 15 min): *t*_R_ = 5.1 min]. 10 vol% Na-ascorbate solution (100 mM in H_2_O, radiolysis quencher) is added to the resulting product solution. The concentration of [^125^I]I-TOC is determined volumetrically by transferring the solution to a new vessel (20 mL reaction vessel), and the amount of [^125^I]I-TOC contained is measured using an activimeter. The product obtained has a radiochemical yield of RCY (radio-RP-HPLC) = 42.9% and a radiochemical purity of RCP (radio-RP-HPLC) > 99%. Analysis of [^125^I]I-TOC was performed by co-injection of [^nat^I]I-TOC using a radio-RP-HPLC. [^125^I]I-TOC is stored at −4 °C and can be used for up to three weeks.
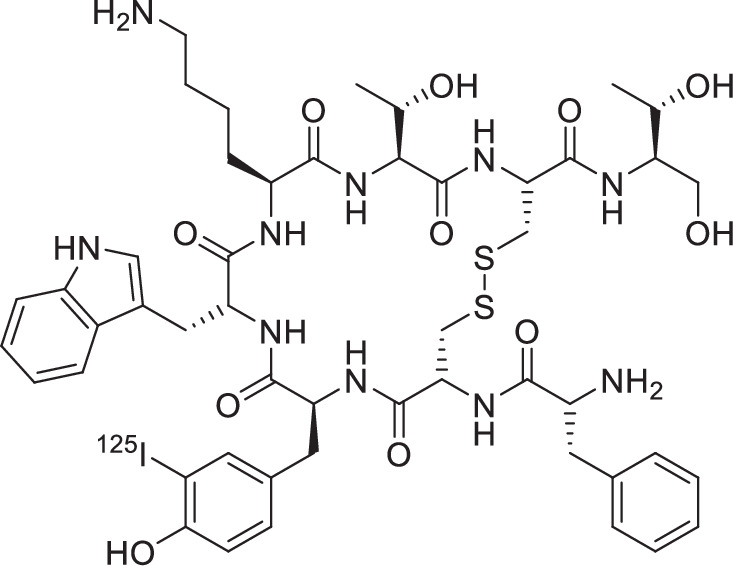


Radio-RP-HPLC (20-50% MeCN/H_2_O with 0.1% TFA, *v*/*v*, 20 min): *t*_R_ = 5.1 min.

### Complexation of the DOTA chelator with non-radioactive lutetium

For the incorporation of lutetium, LuCl_3_ (20 mm in H_2_O, 3.0 eq.) was added to a 2 mm solution of the compound in DMSO and diluted to 1 mm by addition of DMSO. The obtained solution was incubated at 70 °C for 15 min.

### Lipophilicity (log*D*_pH=7.4_)

For the determination of the octanol-PBS partition coefficient (log*D*_*7.4*_ values), 500 µL of 1-octanol and 500 µL of PBS were added to a 1.5 mL reaction tube (Eppendorf Tube^®^) (*n* = 6). Thereafter, 1 MBq of each ^18^F- or ^177^Lu-labeled compound was added and vortexed for 3 min at room temperature. After centrifugation (9000 rpm, 5 min, room temperature), 200 µL of each layer was taken separately, and the activity was quantified by a WIZARD 2480 automatic *γ*-counter (*Perkin Elmer*, Waltham, USA).

### Binding to human serum albumin (HSA)

HSA binding studies were performed according to a previously published procedure, using RP-HPLC and HSA which is solid-phase fixed on a Chiralpak HSA column (50 × 3 mm, 5 μm, H13 h-2433, *Daicel*, Tokyo, Japan)^45^. A flow rate of 0.5 mL/min was used at room temperature. A freshly prepared 50 mm aqueous solution of NH_4_OAc (pH = 6.9) was used as mobile phase A, and isopropanol (HPLC grade, *VWR*, Germany) was used as mobile phase B. A gradient of 100% A (0 to 3 min) followed by 80% A (3 to 40 min) was used for the experiments. Before the analysis of all compounds, a calibration curve with nine reference substances with literature known HSA binding in the range of 13 to 99% was determined (Fig. S20, and Table S2)^45,46^. All compounds, were prepared in a 1/1 mixture (*v*/*v*) of isopropanol and a 50 mm aqueous solution of NH_4_OAc (pH = 6.9) at a final concentration of 0.5 mg/ml. Nonlinear regression was performed using *OriginPro 2016G* software (Northampton, United States).

### Cells and cell culture maintenance

The adherent sstR2-transfected CHO_sstR2_ cells (CHO) cells stably transfected with human sst2R (epitope-tagged at the *N*-terminal end, provided by Dr. Jenny Koenig, University of Cambridge, Cambridge, United Kingdom) were cultured in DMEM/F12 GlutaMax medium (plus 10% FBS *v*/*v*) at 37 °C in a humidified 5% CO_2_ atmosphere. To ensure uniform cell growth, cells were passaged at approximately 80% confluence (2–4 days). The spent medium is removed and the remaining cell lawn washed with PBS (10 mL, 37 °C). By treatment with 5 mL trypsin/EDTA (0.05%/0.02% in PBS without Ca^2+^/Mg^2+^) for 5 min at 37 °C, the cells were detached and suspended by adding 5 mL DMEM/F12 GlutaMax medium (plus 10% FBS *v*/*v*). The suspension was centrifuged (1300 rpm, 3 min, RT) and the cell pellet resuspended in fresh DMEM/F12 GlutaMax medium (20 mL, plus 10% FBS *v*/*v*, 37 °C). A portion of the suspension was transferred to new culture flasks and the volume was adjusted to 25 mL with DMEM/F12 GlutaMax medium (plus 10% FBS *v*/*v*). Cell density was checked regularly under an inverted microscope.

AR42J cells (*CLS GmbH*, Eppelheim, Germany and *Sigma Aldrich*, Gillingham, UK) for ex vivo studies were cultivated in RPMI medium (10% FBS + 2.5 vol% *l*-Gln solution (200 mm) + 1 vol% MEM non-essential amino acid solution, *v*/*v*) at 37 °C in a humidified 5% CO_2_ atmosphere. To ensure uniform cell growth, they were passaged at approximately 80% confluence (2–4 days). The medium was removed and the remaining cell lawn washed with PBS (6 mL, 37 °C). By treatment with EDTA (0.1%) in PBS (5 mL, 5 min, 37 °C), the cells were detached and suspended in 5 mL RPMI medium (10% FBS + 2.5 vol% *l*-Gln solution (200 mM) + 1 vol% MEM non-essential amino acid solution, *v*/*v*). The suspension was centrifuged (1300 rpm, 3 min, RT) and the cell pellet resuspended in fresh RPMI medium (10% FBS + 2.5 vol% *l*-Gln solution (200 mm) + 1 vol% MEM non-essential amino acid solution, *v*/*v*). A portion of the suspension was transferred to new culture flasks and the volume adjusted to 25 mL with RPMI medium (10% FBS + 2.5 vol% *l*-Gln solution (200 mm) + 1 vol% MEM non-essential amino acid solution, *v*/*v*). Cell density was checked regularly under an inverted microscope.

### Receptor binding affinity determinations (IC_50_)

In vitro competition studies were performed on CHO_sstR2_ cells (CHO) cells stably transfected with human sstR2 (epitope-tagged at the *N*-terminal end, provided by Dr. Jenny Koenig, University of Cambridge, Cambridge, United Kingdom), which were seeded (24-well plates, 1.0 × 10^5^ cells/well, DMEM/F12 GlutaMax plus 10% FBS) and incubated at 37 °C for 24 ± 2 h before the experiment. On the day of the experiment, the medium was removed, and each well was washed with 300 µL of HBSS (supplemented with 1 vol% of bovine serum albumin = HBSA). After the addition of 200 µL of HBSA, 25 *µ*L/well of HBSA (control, *n* = 3) or the respective ligand in concentrations ranging from 10^−10^ to 10^−4 ^M (n = 3) was added. Subsequently, 25 µL of the radiolabelled reference [^125^I]I-TOC (1 nm in HBSA, for synthesis and characterization see SI) was added to each well. After incubation at RT for 1 h, the supernatant was removed, washed with ice-cold PBS (300 µL), and the washing solutions were combined with the supernatants. The cells were lysed by adding NaOH (300 µL, 1 m). The cell lysate is removed after incubation at RT for 20 min and washed with NaOH (300 µL, 1 M), while both NaOH-containing fractions were combined. Subsequently, the activities of both the supernatant and the lysate were measured separately in a *γ*-counter, and the *IC*_50_ values (concentration that is needed to replace 50% of the reference competitor from the receptor) were calculated using GraphPad Prism software (*GraphPad Prism 4.0 Software Inc*., La Jolla, California, USA). Data were considered valid when the *R*² fit was > 0.95. Each experiment was performed in triplicate.

### Stability studies in human serum

5 MBq of the respective ^18^F- or ^177^Lu-labeled compound were added to 200 µL of human serum (H4522 from Sigma Aldrich, US) and incubated at 37 °C for 1 h. The human serum supplier certifies that all samples were collected from healthy donors in FDA-licensed facilities following informed consent. After the addition of 50 vol% of cold ethanol and 150 vol% of cold MeCN, centrifugation was performed at 13,000 rpm for 20 min. The supernatant was decanted and centrifuged at 13,000 rpm for 10 min in a centrifuge tube with a 0.45 µm cellulose acetate filter. Approximately 0.2 MBq of the remaining filtrate was injected into RP-HPLC, and the amount of intact radioligand was quantified.

### Ex vivo biodistribution studies

Animal experiments were performed by certified personnel following a previously published method^37^. Experiments were performed in agreement with the general animal welfare regulations in Germany (German Animal Welfare Act, as published on May 18, 2006, as amended by Article 280 of June 19, 2020, permit no. ROB-55.2-2532.Vet_02-18-109 by the *General Directorate of Upper Bavaria*) and institutional guidelines for the care and use of animals. All animal experiments were approved and licensed by the *General Directorate of Upper Bavaria*. Female CD1-nu/nu mice aged 5–6 weeks (*Charles River Laboratories International Inc*., Sulzfeld, Germany) were acclimated in the in-house animal facility for 1 week prior to inoculation. Mice were hold as 2–5 individuals at once in a GM500 cage (T*ecniplast*, Buguggiate, Italy) with a Smartflow AHU ventilation system (T*ecniplast*, Buguggiate, Italy) and adequate diet, bedding and enrichment (*SAFE*, Augy, France). Scoring and other maintenance were performed under an Aria CS60 changing station (T*ecniplast*, Buguggiate, Italy). Inoculation was performed under gas anesthesia with 1–2% isoflurane in oxygen. For this, an isoflurane vaporator Isotec 4 (*Ohmeda/GE healthcare*, Chicago, USA), was used with an anesthetic gas filter Eicksorber (*Eickmeyer KG*, Tuttlingen, Germany). Tumor xenografts were generated using AR42J cells (7.0 × 10^6^ cells per 200 µL) suspended in a 1/1 mixture (*v*/*v*) of RPMI 1640 medium and Cultrex^®^ Basement Membrane Matrix Type 3 (*Trevigen*, Gaithersburg, MD, USA). This suspension was inoculated subcutaneously onto the right shoulder, and animals were used when the tumor volume was >100 mm^3^ (1–2 weeks after inoculation). Exclusion criteria for animals from an experiment were either weight loss greater than 20%, tumor size greater than 1500 mm^3^, tumor ulceration, respiratory distress, or behavioral change. None of these criteria applied to any of the animals from the trial. No randomized or blinded approach was used in the allocation of the experiments. Health status is SPF according to the FELASA recommendation. Biodistribution studies (*n* = 3–5) were performed after 1 h, 6 h, 24 h p.i., and approximately 2–3 MBq (300 pmol) were administered via the tail vein under gas anesthesia with 1–2% isoflurane in oxygen. Euthanazation was performed by stunning with CO_2_ followed by cervical dislocation. Biodistribution was measured by the determination of activity in each organ after tissue separation. Collected data were statistically analyzed using Excel (*Microsoft Corporation*, Redmond, WA, USA) and OriginPro software (version 9.7) from *OriginLab Corporation* (Northampton, MA, USA). In the case of **[**^**177**^**Lu]Lu-rhTATE4**, the study was carried out using a different gamma counter with lower sensitivity. For this reason, measurement limits were determined experimentally and then evaluated graphically, yielding a minimum measurement limit of 97.0 cpm/30 s (see SI, Table S5).

## Supplementary information

Supplementary Information

## Data Availability

All data generated and analyzed in this study are included within the paper and its Supplementary Information. Raw data are available from the corresponding authors on request.
