# Supplementary material for: A sstR2-targeted radiohybrid theranostic agent for PET imaging and β- therapy with excellent preclinical performance
Source: Npj Imaging. 2026 Apr 8;4:24. doi: 10.1038/s44303-026-00155-w (PMC13062086; doi:10.1038/s44303-026-00155-w)
Supplement: Supplementary file 1 — Supplementary Information [file 44303_2026_155_MOESM1_ESM.pdf]

Supplementary Information

A sstR2-targeted radiohybrid theranostic agent for PET imaging and β^- therapy with excellent preclinical performance

Sandra Deiser[1,2,§], Sebastian Fenzl[1,3,§], Victor König[1], Shigeyoshi Inoue[3,*] and Angela Casini[1,2,*]

[1] *Chair of Pharmaceutical Radiochemistry, Department of Chemistry, School of Natural Sciences, Technical University of Munich, Walther-Meißner-Str. 3, 85748 Garching b. München, Germany*

[2] *Chair of Medicinal and Bioinorganic Chemistry, Department of Chemistry, TUM School of Natural Sciences, Technical University of Munich, Lichtenbergstr. 4, 85748 Garching b. München, Germany*

[3] *Chair of Silicon Chemistry, School of Natural Sciences, Technical University of Munich, Lichtenbergstr. 4, 85748 Garching b. München, Germany*

[§] Equal contribution

*Corresponding authors: angela.casini@tum.de; s.inoue@tum.de

List of Abbreviations

To simplify the nomenclature of the SST-ligands, the three-letter code was used to describe the amino acids.

2-CTC	2-Chlorotrityl chloride
DCM	Dichloromethane
DIPEA	*N*,*N*-diisopropylethylamine
DMEM	Dulbecco's modified Eagle's medium
DMF	Dimethylformamide
DMSO	Dimethyl sulfoxide
DOTA(^{t}Bu)$_3$	1,4,7,10-Tetraazacyclododecane-1,4,7,10-tetraacetic acid 1,4,7-tri-tert-butyl
DOTA(^{t}Bu)$_2$	*trans*-(di-*tert*-butyl)-1,4,7,10-tetraazacyclododecane-1,4,7,10-tetraacetic acid
DOTA-GA	2-[1,4,7,10-Tetraazacyclododecane]-pentanedioic acid
ESI-MS	Electrospray ionization mass spectrometry
FBS	Fetal bovine serum
Fmoc	Fluorenylmethoxycarbonyl
HATU	Hexafluorophosphate azabenzotriazole tetramethyl uronium
HBSA	Hank's balanced salt solution + 1% bovine serum albumine
HBSS	Hank's balanced salt solution
HOAt	1-Hydroxy-7-azabenzotriazole
HSA	Human serum albumin
IC_{50}	Half maximal inhibitory concentration
log$D_{7.4}$	Octanol-PBS$_{7.4}$ partition coefficient
MeCN	Acetonitrile
MeOH	MeOH
NMP	1-Methylpyrrolidin-2-one
O$_2$Oc	8-Amino-3,6-dioxaoctanoic acid
PBS	Phosphate-buffered saline
RCC	Radiochemical conversion
RCP	Radiochemical purity
RCY	Radiochemical yield
RP-HPLC	Reverse phase high performance liquid chromatography
SPPS	Solid-phase peptide synthesis
sst	Somatostatin
t_R	Retention time
TBTU	2-(1-H-Benzotriazole-1-yl)-1,1,3,3-tetramethylaminium tetrafluoroborate
TFA	Trifluoroacetic acid
TIPS	Triisopropylsilane

Figures

HPLC chromatograms

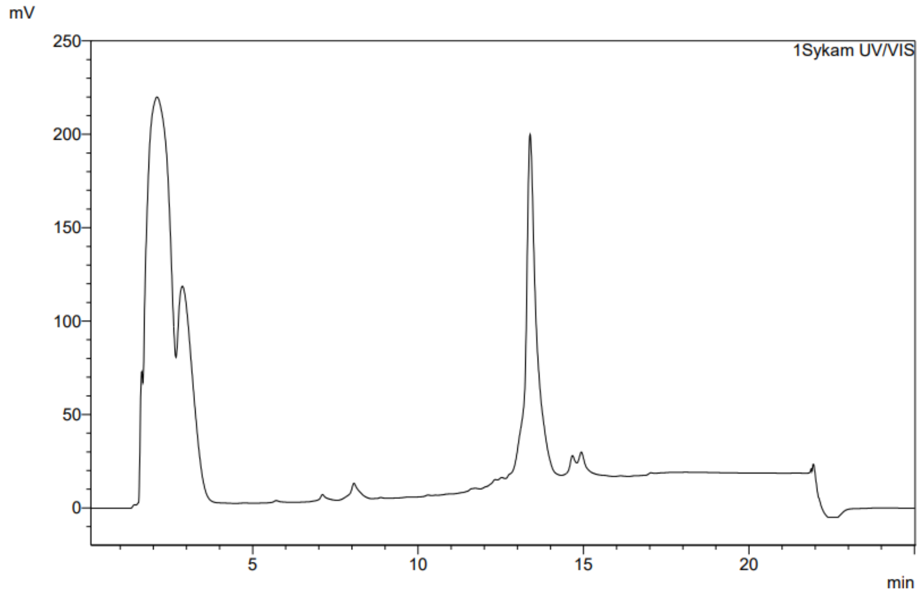

Figure S1: RP-HPLC chromatogram of the reaction control of H-TATE(PG)-OH using the analytical control method 10-90% B (15 min, λ = 220 nm, MultoKrom® 100-5 C18-column (125 × 4.6 mm, 5 µm particle size, CS Chromatographie GmbH, t_R = 13.4 min).

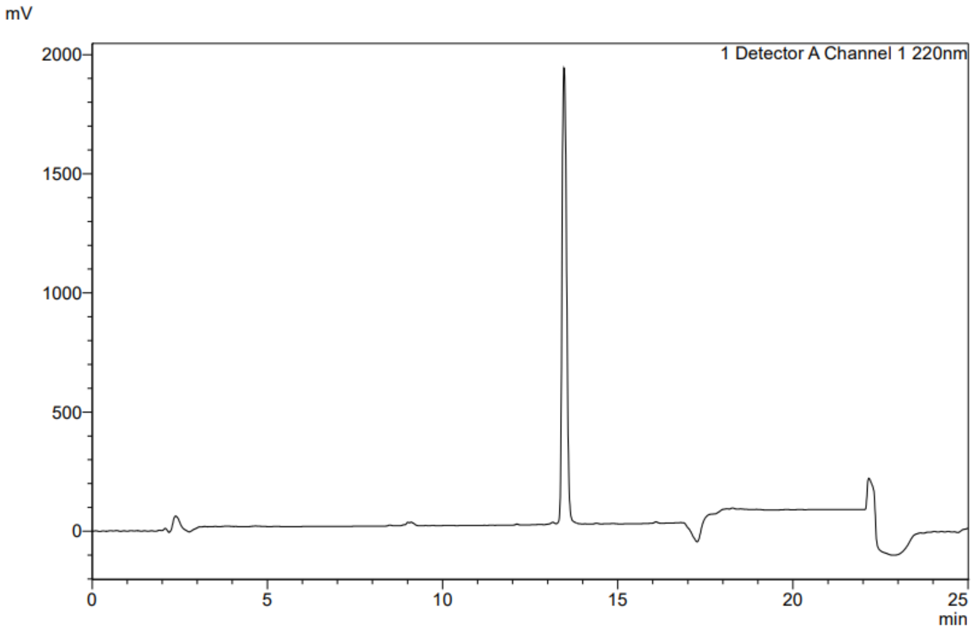

Figure S2: RP-HPLC chromatogram of SiFA*lin*-TATE using the analytical control method 10-60% B (15 min, λ = 220 nm, MultoKrom® 100-5 C18-column (125 × 4.6 mm, 5 µm particle size, CS Chromatographie GmbH), t_R = 13.5 min).

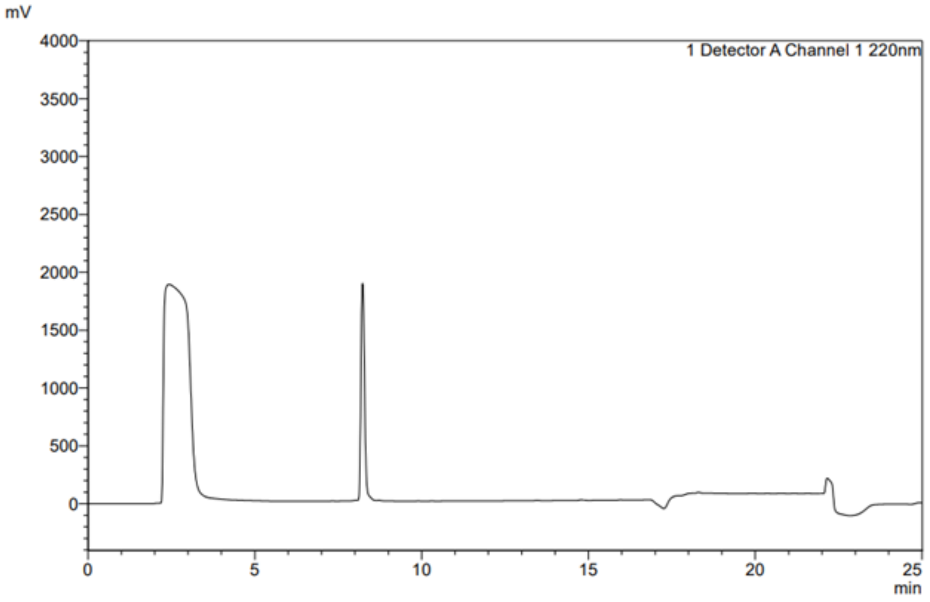

Figure S3: RP-HPLC chromatogram of Lu-DOTA-TATE using the analytical control method 10-60% B (15 min, $\lambda = 220$ nm, MultoKrom® 100-5 C18-column (125 × 4.6 mm, 5 µm particle size, CS Chromatographie GmbH), $t_R = 8.5$ min).

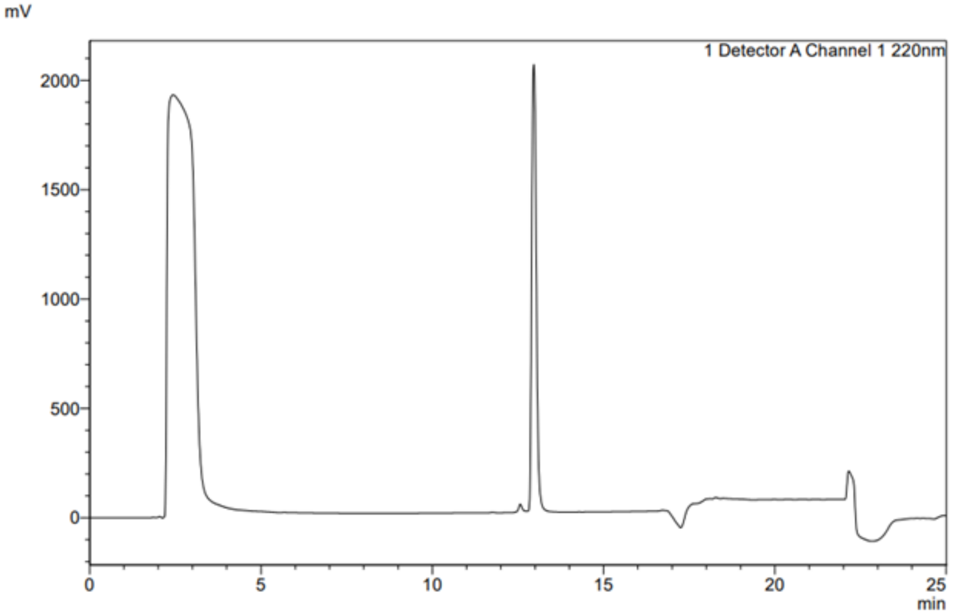

Figure S4: RP-HPLC chromatogram of **Lu-rhTATE4** using the analytical control method 10-60% B (15 min, $\lambda = 220$ nm, MultoKrom® 100-5 C18-column (125 × 4.6 mm, 5 µm particle size, CS Chromatographie GmbH), $t_R = 13.0$ min).

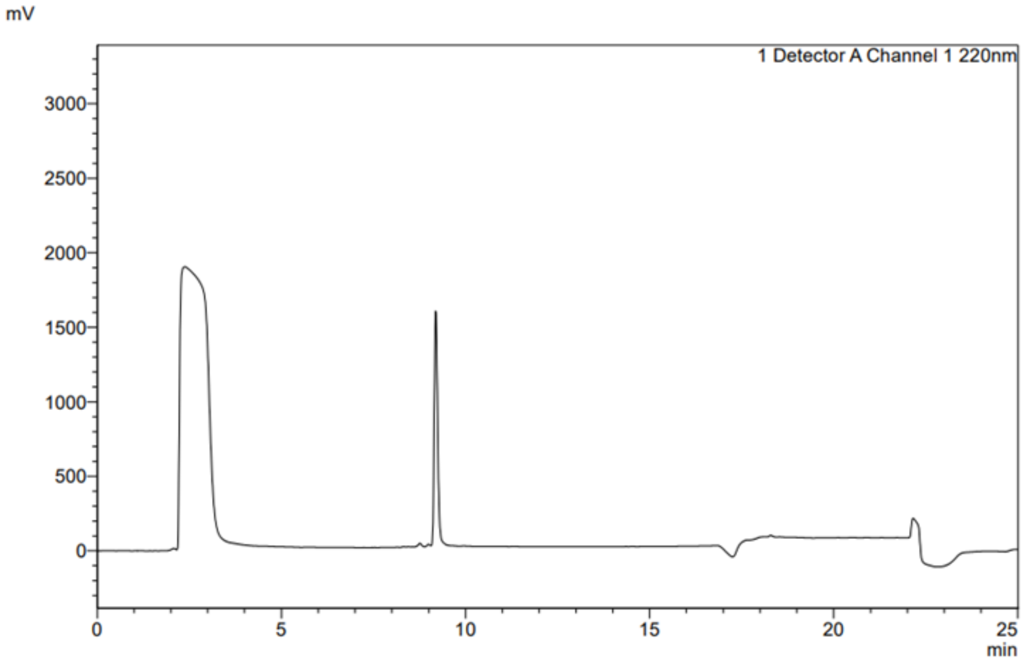

Figure S5: RP-HPLC chromatogram of [^{nat}I]TOC using the analytical control method 10-60% B (15 min, λ = 220 nm, MultoKrom® 100-5 C18-column (125 × 4.6 mm, 5 µm particle size, CS Chromatographie GmbH), t_R = 9.2 min).

Mass spectra

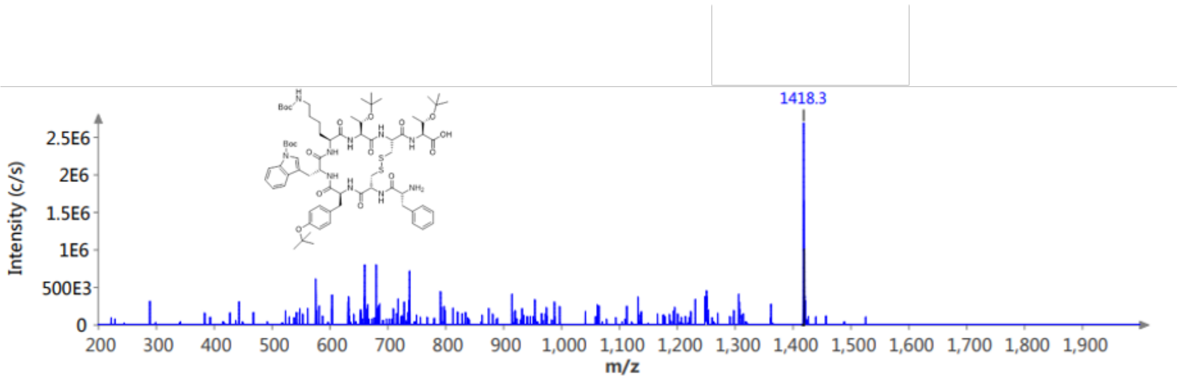

Figure S6: ESI$^+$-mass spectrum of H-TATE(PG)-OH.

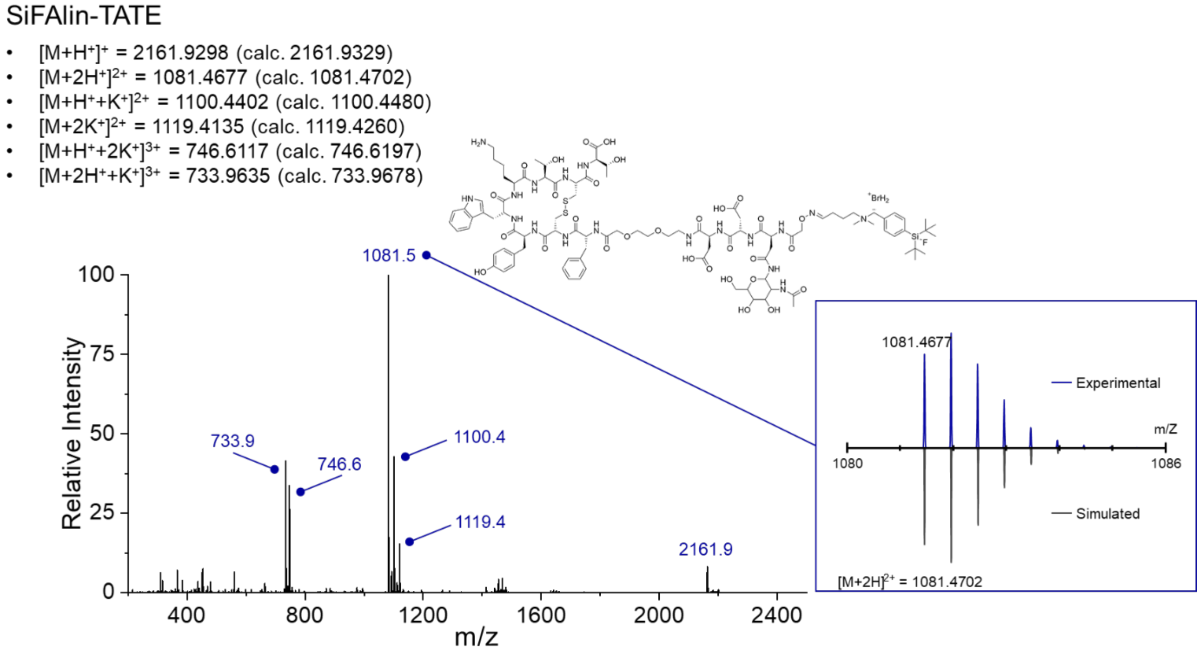

Figure S7: HR-ESI$^+$ mass spectrum of SiFA*lin*-TATE.

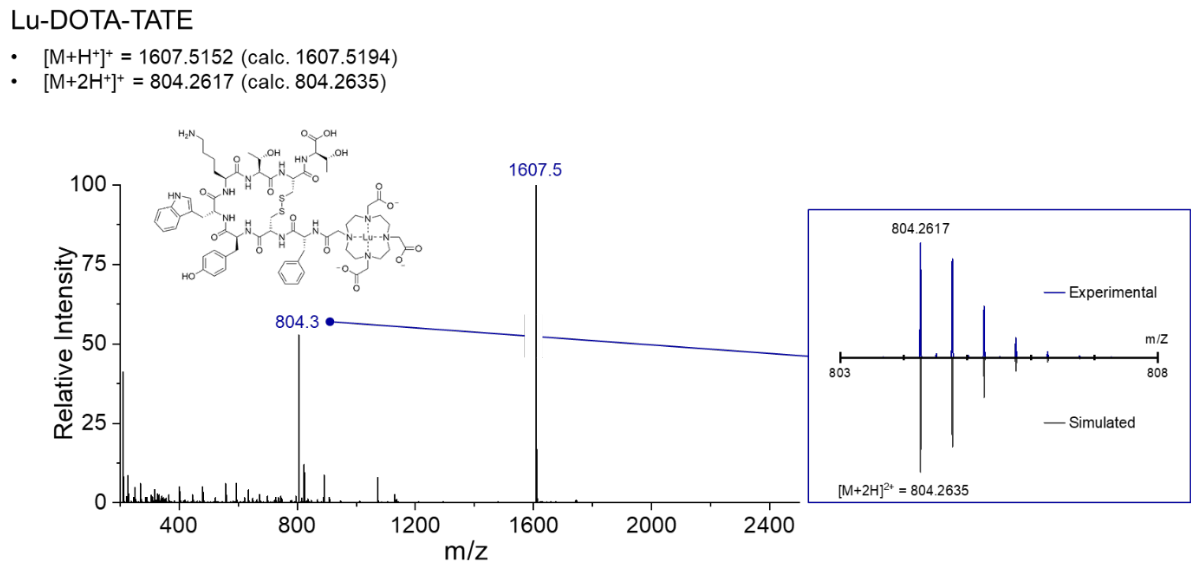

Figure S8: HR-ESI$^+$ mass spectrum of Lu-DOTA-TATE.

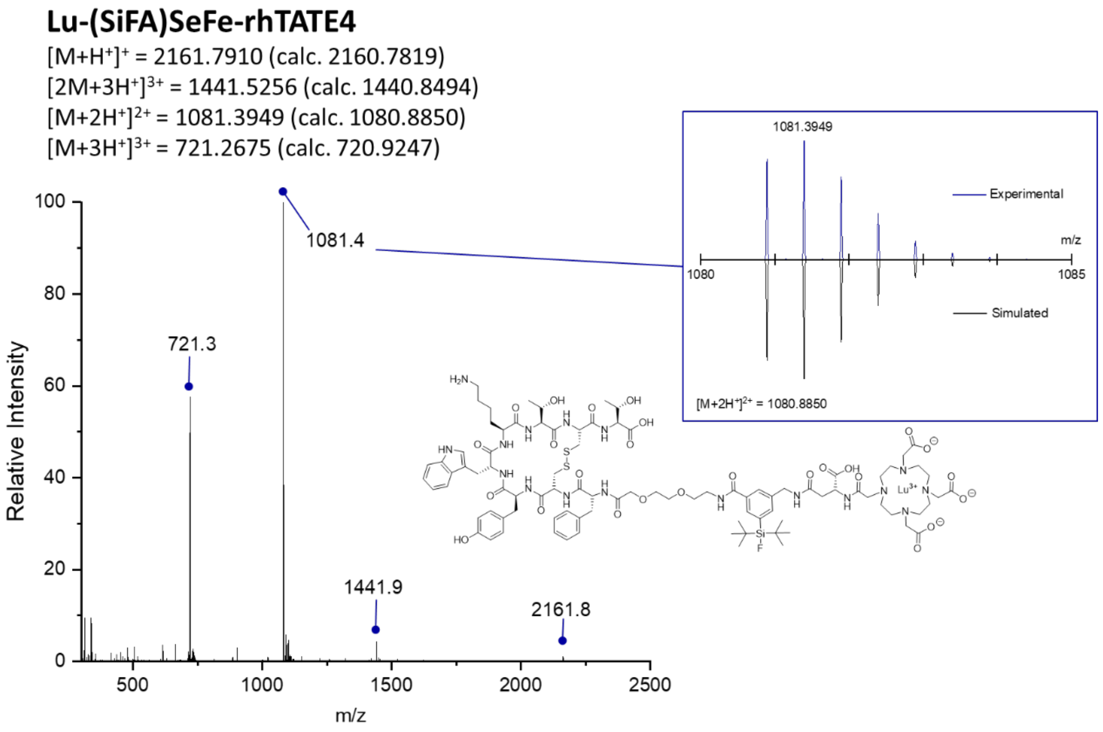

Figure S9: HR-ESI$^+$ mass spectrum of **Lu-rhTATE4**.

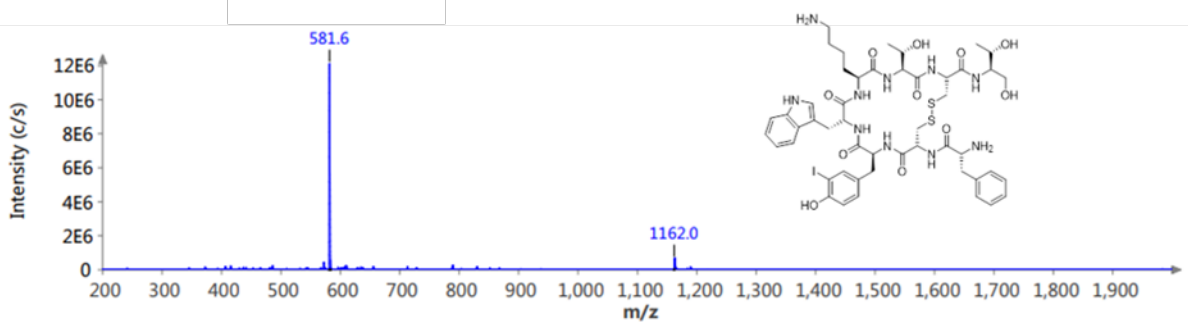

Figure S10: ESI mass spectrum of I-TOC.

Radiochemical characterization

Radio-RP-HPLC

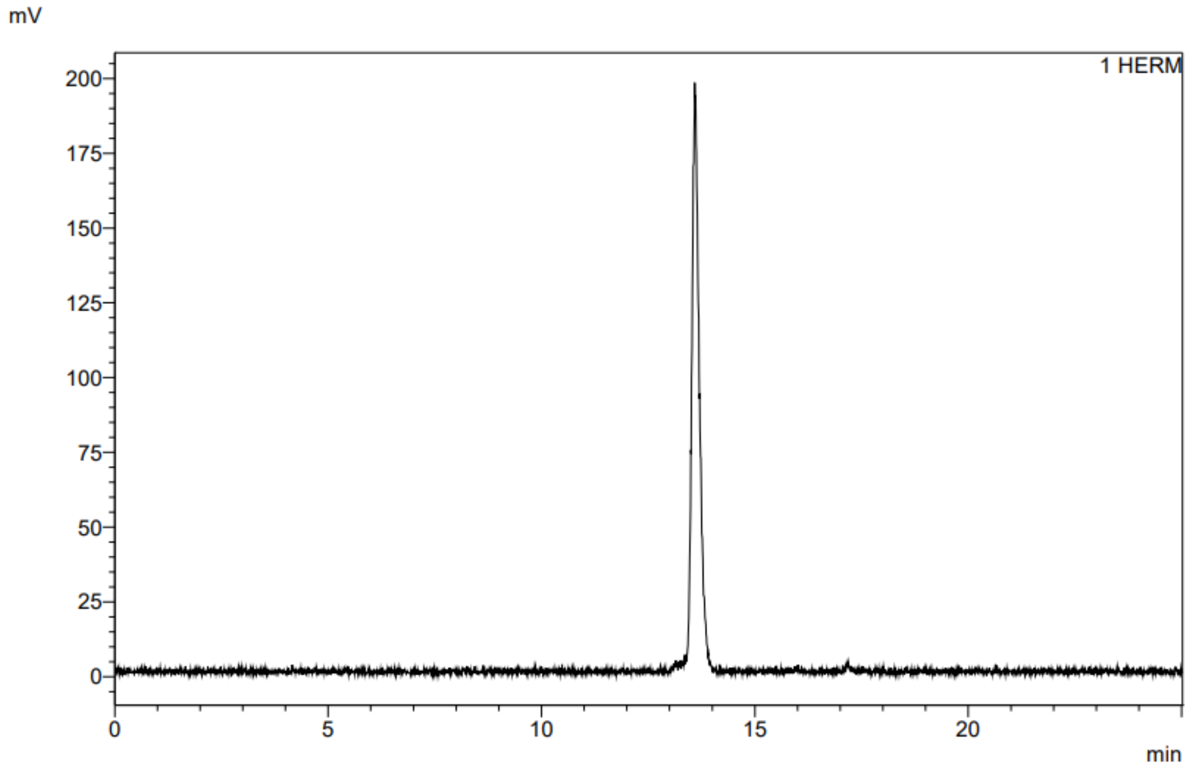

Figure S11: Radio-RP-HPLC chromatogram of [^{18}F]SiFA*lin*-TATE using the analytical control method 10-60% B (15 min, MultoKrom® 100-5 C18-column (125 × 4.6 mm, 5 µm particle size, CS Chromatographie GmbH), t_R = 13.6 min).

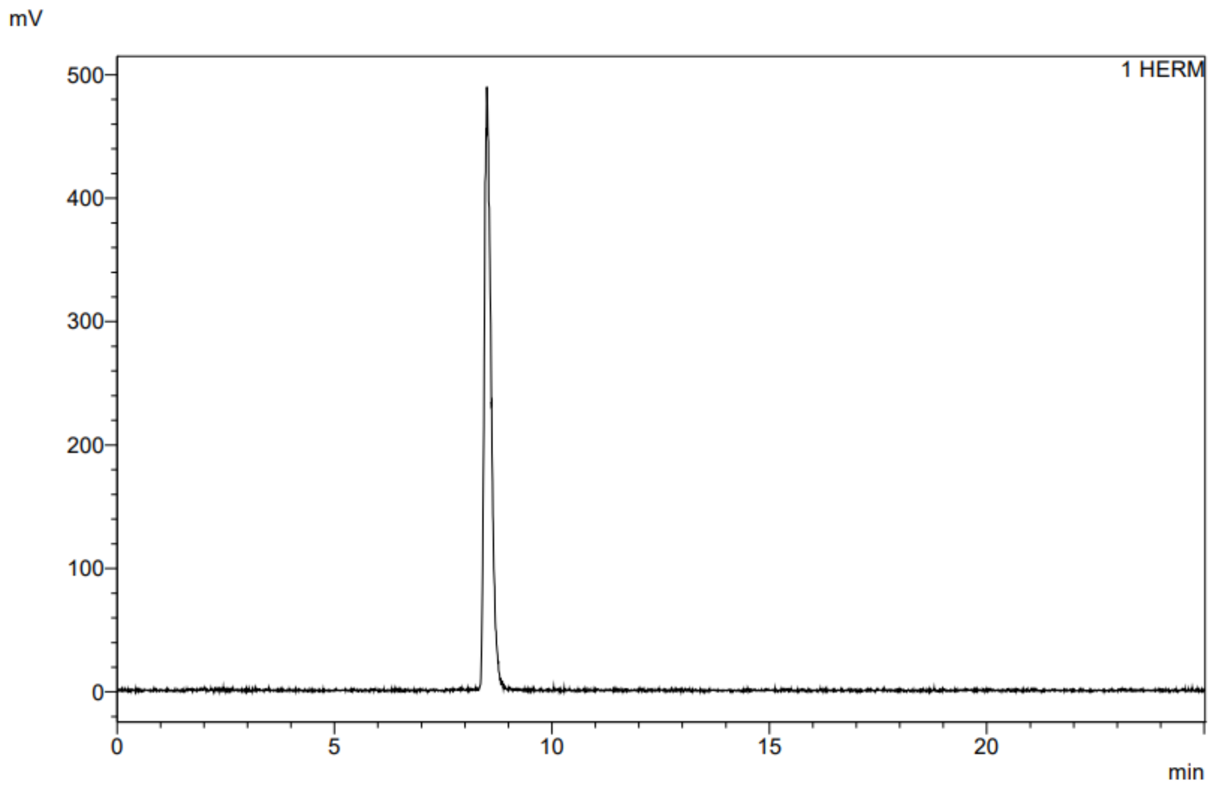

Figure S12: Radio-RP-HPLC chromatogram of [^{177}Lu]Lu-DOTA-TATE using the analytical control method 10-60% B (15 min, MultoKrom® 100-5 C18-column (125 × 4.6 mm, 5 µm particle size, CS Chromatographie GmbH), t_R = 8.5 min).

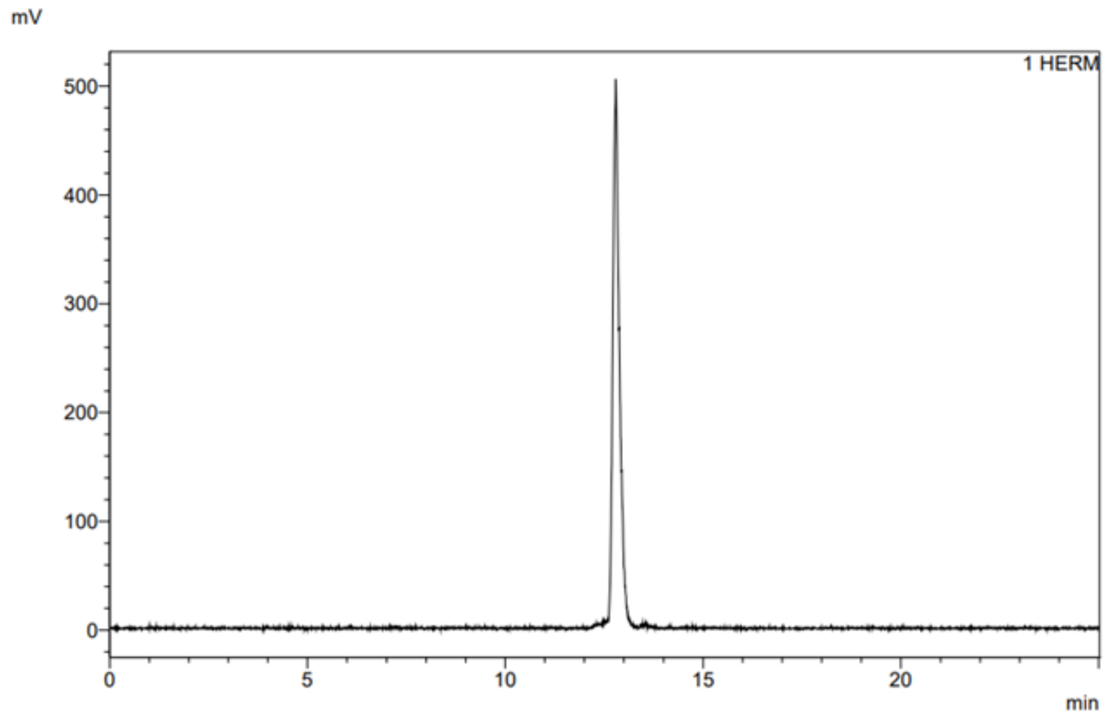

Figure S13: Radio-RP-HPLC chromatogram of **[^{18}F]Lu-rhTATE4** using 10-60% B (125 × 4.6 mm, 5 µm particle size, CS Chromatographie GmbH), t_R = 14.6 min)**.**

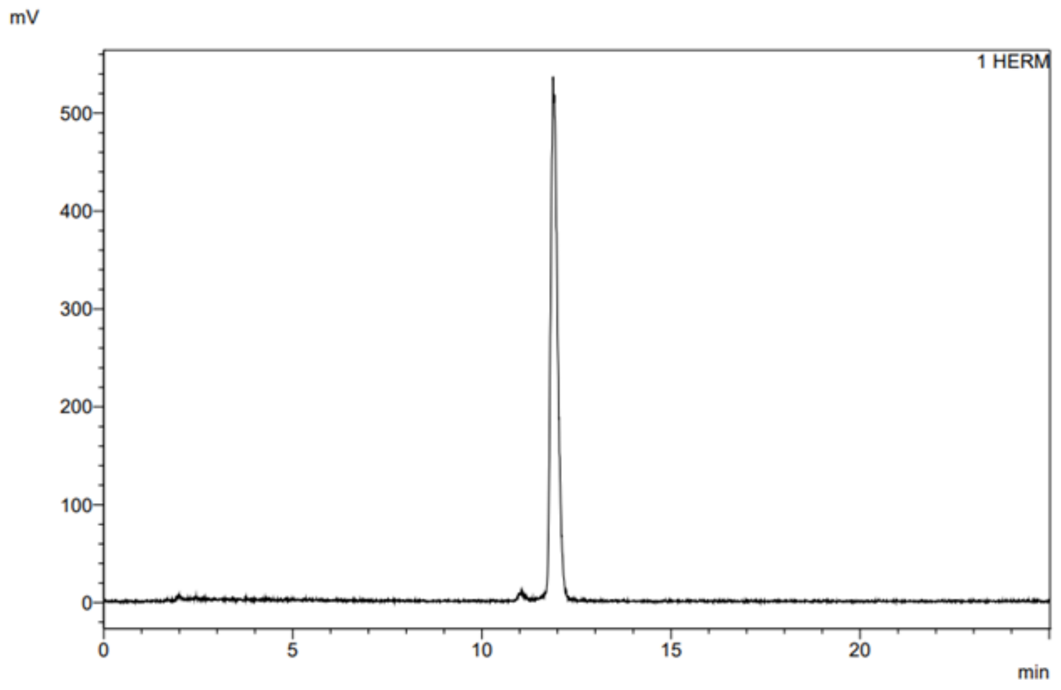

Figure S14: Radio-RP-HPLC chromatogram of **[^{177}Lu]Lu-rhTATE4** using 10-60% B (125 × 4.6 mm, 5 µm particle size, CS Chromatographie GmbH), t_R = 11.9 min)**.**

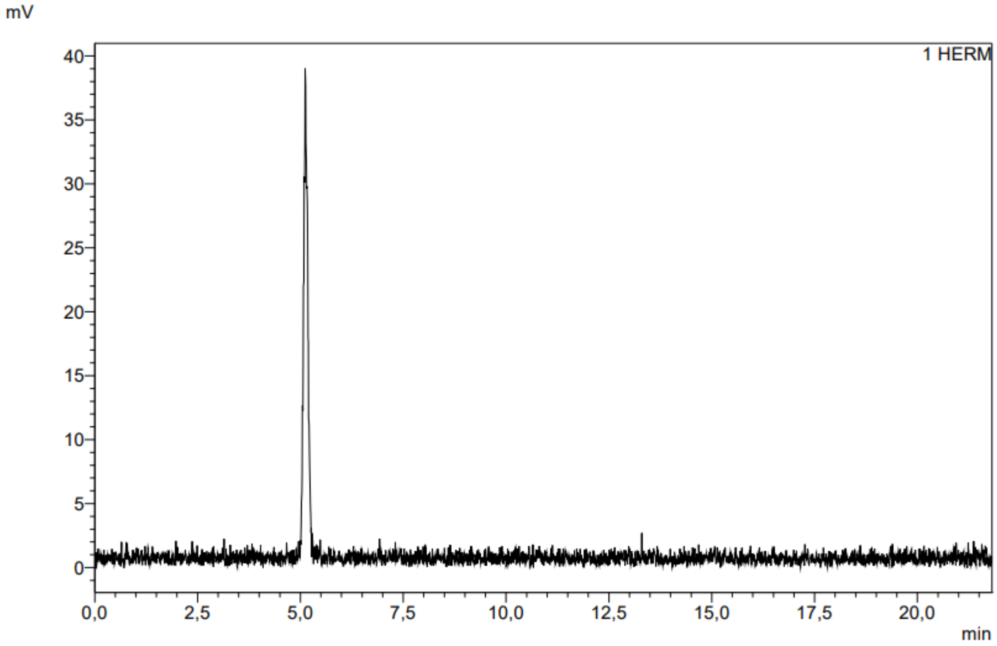

Figure S15: Radio-RP-HPLC chromatogram of [^{125}I]TOC using the analytical control method 20-50% B (15 min, MultoKrom® 100-5 C18-column (125 × 4.6 mm, 5 µm particle size, CS Chromatographie GmbH), t_R = 5.1 min).

Radio-TLC

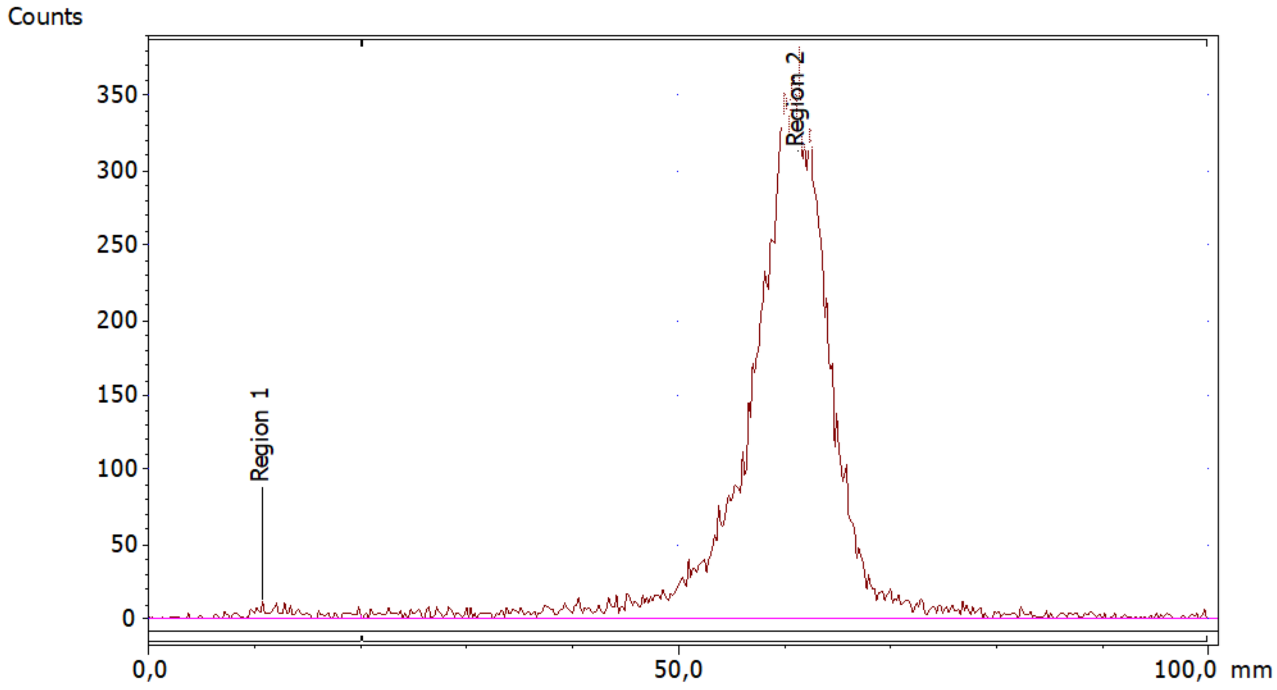

Figure S16: Radio-TLC chromatogram of [^{18}F]SiFA*lin*-TATE (flow agent: 60% MeCN/ 40% PBS (6/4 *v*/*v*) with 10% NaOAc in H_2O (2 M) and 1% TFA, stationary phase: TLC silica gel 60 F_{254} from Merck Millipore).

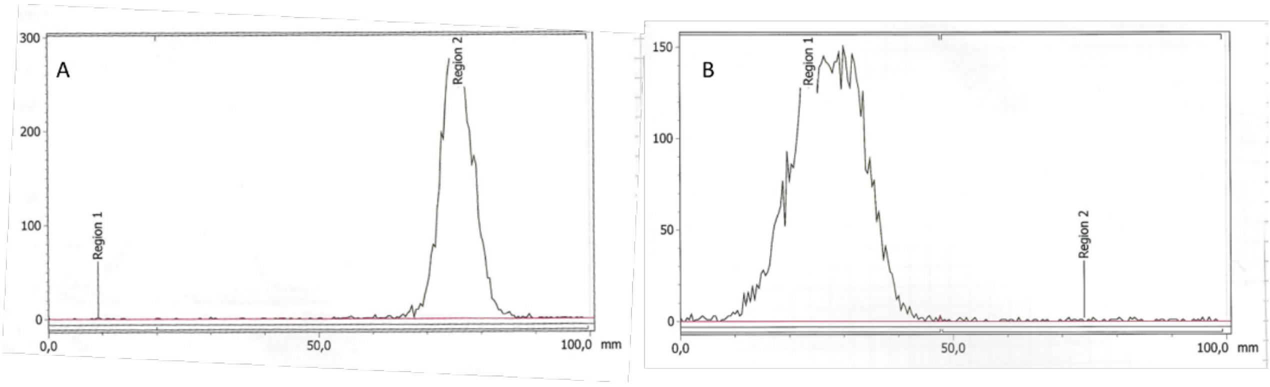

Figure S17: Radio-TLC chromatogram of [^{177}Lu]Lu-DOTA-TATE using A: 1 M NH_4OAc/DMF (1/1, *v*/*v*), stationary phase: TLC silica gel 60 F_{254} and B: 0.1 M sodium citrate × 1.5 H_2O, stationary phase: iTLC-SC.

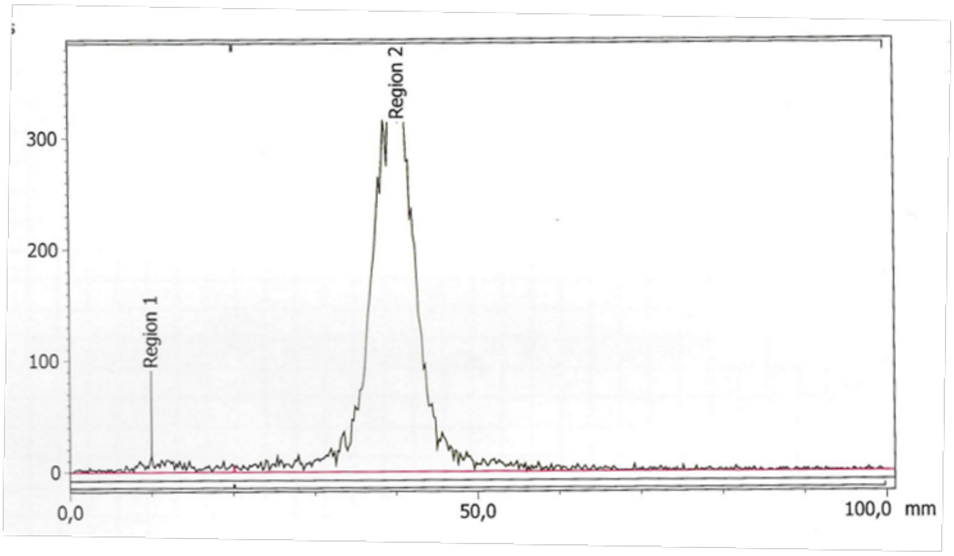

Figure S18: Radio-TLC chromatogram of [^{18}F]Lu-rhTATE4 using 60% MeCN/ 40% PBS (6/4 *v*/*v*) with 10% NaOAc in H_2O (2 M) and 1% TFA, stationary phase: TLC silica gel 60 F_{254} from Merck Millipore.

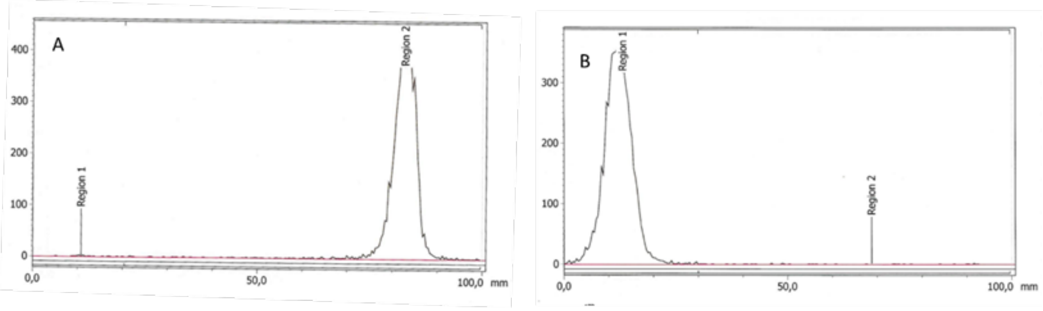

Figure S19: Radio-TLC chromatogram of **[^{177}Lu]Lu-rhTATE4** using A: 1 M NH_4OAc/DMF (1/1, *v*/*v*), stationary phase: TLC Silica gel 60 F_{254} and B: 0.1 M sodium citrate × 1.5 H_2O, stationary phase: iTLC-SC.

In vitro and *in vivo* data

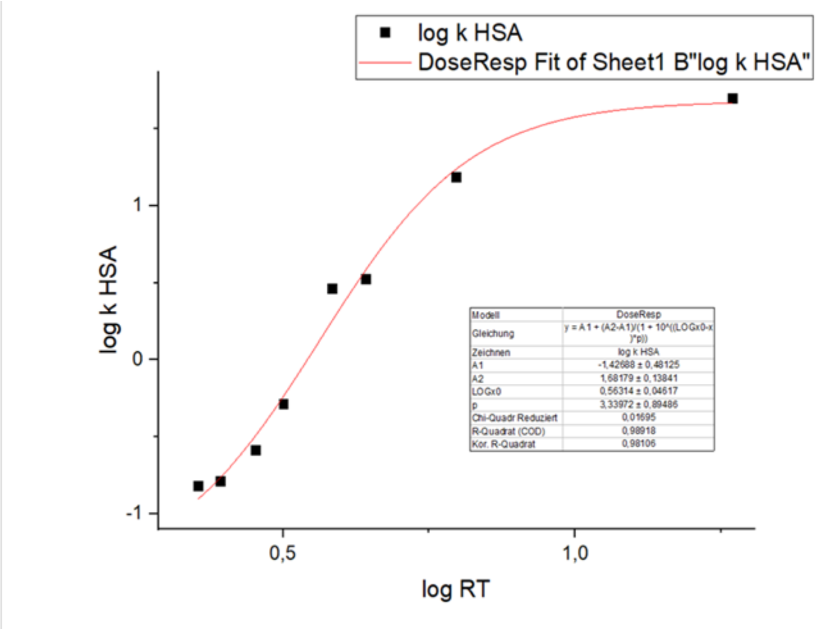

Figure S20: Fit function calculated by non-linear regression to determine the HSA binding.

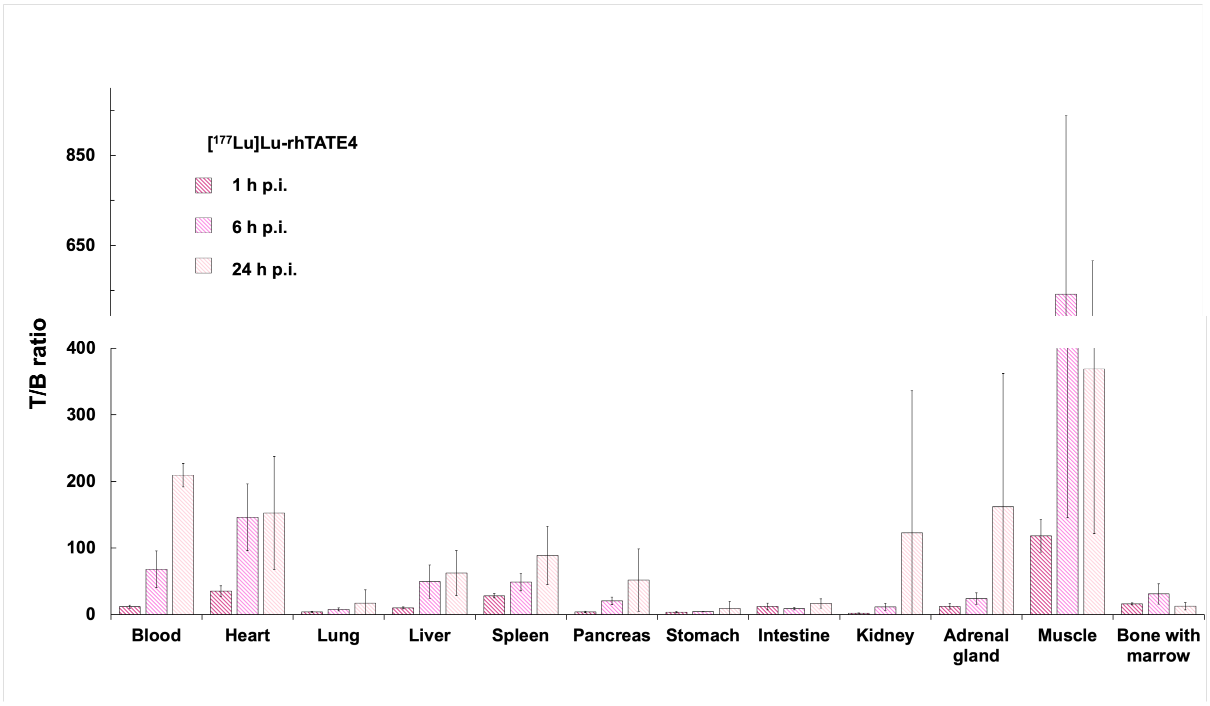

Figure S21: T/B-ratios of **[^{177}Lu]Lu-rhTATE4** at 1 h, 6 h and 24 h p.i. (300 pmol each).

Tables

Table S1: Deviating equivalents, coupling reagents, base (DIPEA), pre-activation times, and reaction times for coupling the respective substrates to the resin-bound peptide (1.0 eq.).

Substrate	eq.	TBTU [eq.]	HATU [eq.]	HOAt [eq.]	Base [eq.]	Preactivation [min]	Time [h]
Fmoc-O_2Oc-OH	2.0	-	1.9	1.9	2.0	15	1.5
DOTA(*t*Bu)$_3$	1.5	-	1.5	1.5	4.5	15	3.0
Fmoc-*L*-Asp(^{t}Bu)-OH	3.0	3.0	-	3.0	9.0	2	2.0
Fmoc-Asn(Ac_3AcNH-*β*-Glc)-OH	2.0	1.9	1.9	-	2.0	2	2.0
bis-Boc-amino-oxyacetic acid	2.0	1.9	-	1.9	2.0	2	2.0
Fmoc-(SiFA)*SeFe*-OH	1.5	1.5	-	1.5	3.5	15	2.0

Table S2: Compounds for the calibration curve and their respective literature-known HSA-binding percentages.[2-3]

**Compound**	**HSA-binding [%]**
Benzyl alcohol	13.15
Aniline	14.06
Phenol	20.69
Benzoic acid	34.27
Carbamazepine	75.00
4-Nitrophenol	77.65
Estradiol	94.81
Probenecid	95.00
Glibenclamide	99.00

Nonlinear regression was performed using *OriginPro 2016G* software. (Northampton, United States) using a fit function to calculate the HSA bindings of the examined compounds based on their retention time.

Table S3: Results of ^{18}F-/^{177}Lu-labelling reporting radiochemical conversion (RCC), radiochemical yield decay corrected ($RCY_{d.c.}$), radiochemical purity *via* radio-RP-HPLC (RCP_{HPLC}), and radiochemical purity *via* radio-TLC (RCP_{TLC}).

Compound	RCC [%]	$RCY_{d.c.}$ [%]	RCP_{HPLC} [%]	RCP_{TLC} [%]
[^{18}F]SiFA*lin*-TATE	59	59	98	98
[^{177}Lu]Lu-DOTA-TATE	-	≥99	≥99	≥99
[^{18}F]Lu-rhTATE4	55	38	98	99
**[^{177}Lu]Lu-rhTATE4**	-	99	99	99

Table S4: Biodistribution of **[$^{nat/18}$F]Lu-rhTATE4** in selected organs [%ID/g] at 1 h, 6 h and 24 h p.i. in AR42J tumor-bearing female CD1-nu/nu mice. * Out of linear regression of the device.

Organ	^{18}F-labelled		^{177}Lu-labelled		
	1 h (n=3)	1 h block (n=1)	1 h (n=5)	6 h (n=6)	24 h (n=5)
Blood	4.88 ± 0.23	6.13	2.70 ± 0.38	0.32 ± 0.13	0.04 ± 0.02
Heart	1.38 ± 0.08	1.57	0.91 ± 0.13	0.15 ± 0.05*	0.05 ± 0.02
Lung	6.87 ± 0.21	6.34	7.57 ± 0.93	3.37 ± 1.68	1.30 ± 0.68
Liver	5.47 ± 0.47	6.26	3.11 ± 0.41	0.53 ± 0.28	0.19 ± 0.09
Spleen	1.34 ± 0.08	1.27	1.17 ± 0.39	0.49 ± 0.29	0.16 ± 0.10
Pancreas	11.65 ± 0.51	0.75	8.66 ± 3.93	1.05 ± 0.37	0.26 ± 0.18
Stomach	10.63 ± 0.99	2.50	9.30 ± 2.43	5.08 ± 2.43	2.14 ± 0.98
Intestine	2.50 ± 0.20	1.83	3.14 ± 1.50	2.83 ± 1.52	0.63 ± 0.43
Kidney	26.88 ± 1.49	40.30	14.80 ± 1.95	1.93 ± 0.70	0.55 ± 0.26
Adrenal gland	3.27 ± 0.39	1.98	2.67 ± 0.52*	0.93 ± 0.38*	0.22 ± 0.14
Muscle	0.34 ± 0.03	0.50	0.27 ± 0.06*	0.07 ± 0.06*	0.01 ± 0.01
Bone + marrow	1.74 ± 0.45	1.56	1.97 ± 0.43*	0.77 ± 0.45*	1.31 ± 0.68
Bone	0.56 ± 0.29	0.89	-	-	-
Tumor	53.58 ± 5.51	1.96	32.15 ± 8.55	22.97 ± 12.04	10.32 ± 7.04

Table S5: T/B ratio of **[^{18}F]Lu-rhTATE4** and **[^{177}Lu]Lu-rhTATE4** in selected organs [%ID/g] at 1 h, 6 h and 24 h p.i. in AR42J tumor-bearing female CD1-nu/nu mice. * Out of linear regression of the device.

Organ	^{18}F-labelled	^{177}Lu-labelled		
	1 h (n=3)	1 h (n=5)	6 h (n=6)	24 h (n=5)
Blood	10.87 ± 0.78	11.82 ± 2.52	68.35 ± 27.52	209.44 ± 17.56
Heart	38.49 ± 5.26	35.26 ± 7.94	146.24 ± 50.23*	152.49 ± 84.74
Lung	7.37 ± 0.97	4.20 ± 0.83	7.80 ± 2.28	17.24 ± 20.16
Liver	9.09 ± 1.73	10.15 ± 1.61	49.52 ± 24.92	62.47 ± 33.76
Spleen	40.13 ± 4.45	28.36 ± 3.14	48.85 ± 13.13	89.06 ± 43.70
Pancreas	4.54 ± 0.54	4.17 ± 1.24	20.17 ± 5.60	51.80 ± 46.97
Stomach	4.86 ± 0.26	3.67 ± 1.23	4.48 ± 0.55	9.38 ± 10.45
Intestine	21.31 ± 3.54	12.34 ± 5.11	8.96 ± 2.00	16.79 ± 6.98
Kidney	1.85 ± 0.26	2.14 ± 0.38	11.58 ± 5.41	122.78 ± 213.46
Adrenal gland	17.33 ± 1.73	12.44 ± 4.32*	24.09± 8.58*	162.17 ± 200.05
Muscle	143.62 ± 24.52	118.32 ± 24.68*	542.02 ± 396.48*	368.79 ± 247.24
Bone + marrow	31.61 ± 6.12	16.24 ± 1.60*	31.16 ± 15.29*	12.66 ± 5.43
Bone	77.52 ± 27.54	-	-	-

Table S6: Statistical analysis (one-sided, homoscedastic t-test, α = 0.05) of biodistribution data of **[^{177}Lu]Lu-rhTATE4** 1 h vs. 6 h vs. 24 h p.i.. Statistically equal measurements are highlighted in red.

	p		
	1 h vs. 6 h	6 h vs. 24 h	1 h vs. 24 h
**Blood**	1,21083E-06	0,00146999	3,40815E-07
**Heart**	1,78023E-06	0,00408834	4,38749E-07
**Lung**	0,001188107	0,02597755	2,23301E-06
**Liver**	3,15121E-06	0,02646837	3,23565E-07
**Spleen**	0,0118721	0,03279576	0,000496989
**Pancreas**	0,002416856	0,00230711	0,001355782
**Stomach**	0,019573007	0,02771209	0,000296302
**Intestine**	0,386839659	0,01189164	0,006110924
**Kidney**	8,15925E-07	0,00297394	2,51272E-07
**Adrenal gland**	0,000319941	0,0039763	8,62009E-06
**Muscle**	0,000693088	0,06925487	1,25504E-05
**Bone with marrow**	0,002554383	0,11071676	0,071711212
**Tumor**	0,124603107	0,05360497	0,002146877

Table S7: Statistical analysis (one-sided, homoscedastic t-test, α = 0.05) of biodistribution data of **[^{18}F]Lu-rhTATE4** vs **[^{177}Lu]Lu-rhTATE4** 1 h p.i.. Statistically equal measurements are highlighted in red.

	p
**Blood**	0,000187492
**Heart**	0,001672095
**Lung**	0,202418983
**Liver**	0,000175509
**Spleen**	0,332711931
**Pancreas**	0,171245143
**Stomach**	0,23585362
**Intestine**	0,255939703
**Kidney**	6,36297E-05
**Adrenal gland**	0,221846597
**Muscle**	0,040088734
**Bone with marrow**	0,254438875
**Tumor**	0,011468952

Table S8. Statistical analysis (one-sided, homoscedastic t-test, α = 0.05) of biodistribution data of **[^{18}F]Lu-rhTATE4** at 1 h p.i. with respect to previously reported compounds **[^{18}F]-rhTATE1/3** at 1 h p.i., Statistically equal measurements are highlighted in red.

	**p**	
	**[^{18}F]rhTATE1**	**[^{18}F]rhTATE3**
**Blood**	4,92686E-05	4,7625E-05
**Heart**	0,000435959	0,00344151
**Lung**	0,329475525	0,0287543
**Liver**	0,000701675	0,02618696
**Spleen**	0,003698056	0,1734702
**Pancreas**	0,022797307	0,02118465
**Stomach**	0,127561221	0,0699453
**Intestine**	0,31692714	0,0141307
**Kidney**	0,185625702	0,00010459
**Adrenal gland**	0,48986574	0,10838051
**Muscle**	0,000509813	0,47835613
**Bone with marrow**	0,14194309	0,19678766
**Tumor**	0,008788302	0,01532121
